# Vav independently regulates synaptic growth and plasticity through distinct actin-based processes

**DOI:** 10.1083/jcb.202203048

**Published:** 2022-08-17

**Authors:** Hyun Gwan Park, Yeongjin David Kim, Eunsang Cho, Ting-Yi Lu, Chi-Kuang Yao, Jihye Lee, Seungbok Lee

**Affiliations:** 1 Department of Brain and Cognitive Sciences, Seoul National University, Seoul, Korea; 2 Institute of Biological Chemistry, Academia Sinica, Taipei, Taiwan; 3 Department of Oral Pathology, School of Dentistry, Pusan National University, Yangsan, Korea; 4 Department of Cell and Developmental Biology and Dental Research Institute, Seoul National University, Seoul, Korea

## Abstract

Modulation of presynaptic actin dynamics is fundamental to synaptic growth and functional plasticity; yet the underlying molecular and cellular mechanisms remain largely unknown. At *Drosophila* NMJs, the presynaptic Rac1-SCAR pathway mediates BMP-induced receptor macropinocytosis to inhibit BMP growth signaling. Here, we show that the Rho-type GEF Vav acts upstream of Rac1 to inhibit synaptic growth through macropinocytosis. We also present evidence that Vav-Rac1-SCAR signaling has additional roles in tetanus-induced synaptic plasticity. Presynaptic inactivation of Vav signaling pathway components, but not regulators of macropinocytosis, impairs post-tetanic potentiation (PTP) and enhances synaptic depression depending on external Ca^2+^ concentration. Interfering with the Vav-Rac1-SCAR pathway also impairs mobilization of reserve pool (RP) vesicles required for tetanus-induced synaptic plasticity. Finally, treatment with an F-actin–stabilizing drug completely restores RP mobilization and plasticity defects in *Vav* mutants. We propose that actin-regulatory Vav-Rac1-SCAR signaling independently regulates structural and functional presynaptic plasticity by driving macropinocytosis and RP mobilization, respectively.

## Teaser

Park, Kim, Cho, et al. show that actin-regulatory Vav-Rac1-SCAR signaling independently regulates structural and functional presynaptic plasticity by driving BMP macropinocytosis and degradation of BMP receptors and mobilization of the reserve synaptic-vesicle pool, respectively. This work suggests that Vav differentially regulates distinct presynaptic actin pools with highly specialized tasks.

## Introduction

Synapses are highly dynamic structures that undergo structural and functional changes in response to genetic programs and environmental cues. This plasticity is thought to underlie neural circuit refinement during development and higher brain functions (e.g., learning and memory) in adults. The glutamatergic neuromuscular junction (NMJ) in *Drosophila* has served as a useful model for dissecting molecular mechanisms that regulate synaptic growth and activity-dependent plasticity (Frank et al., 2020; Menon et al., 2013). *Drosophila* NMJ synapses initially form during embryogenesis and continuously expand during larval development to accommodate the rapidly growing postsynaptic muscles (Schuster et al., 1996). This developmental growth critically depends on the retrograde (muscle to neuron) signal that is defined by the bone morphogenetic protein (BMP) ligand Glass bottom boat (Gbb) secreted from postsynaptic muscles (McCabe et al., 2003). The Gbb signal activates a presynaptic heteromeric complex of type II BMP receptor (BMPR) Wishful thinking (Wit) and either type I BMPR Thickveins (Tkv) or Saxophone (Sax), resulting in the phosphorylation of the receptor-regulated R-Smad Mothers against decapentaplegic (Mad; Aberle et al., 2002; Marques et al., 2002; Rawson et al., 2003). Phosphorylated Mad (P-Mad) then enters the motoneuron nucleus to serve as a transcriptional regulator of synaptic growth.

Apart from promoting synaptic growth, presynaptic BMP signaling also induces macropinocytosis and subsequent intracellular degradation of BMPRs (Kim et al., 2019), preventing excessive BMP signaling to restrict synaptic growth within a normal physiological range. Gbb-induced BMPR macropinocytosis is mediated by the Rho GTPase Rac1 and the SCAR complex (Kim et al., 2019), which transduces Rac1 signaling to trigger Arp2/3-dependent actin nucleation (Mendoza, 2013; Rotty et al., 2013). SCAR protein forms a multimeric complex comprising CYFIP/Sra-1, Kette/Nap1, HSPC300, and Abelson interacting protein (Abi; Derivery et al., 2009; Eden et al., 2002; Gautreau et al., 2004; Lebensohn and Kirschner, 2009). Impairment of presynaptic Rac1-SCAR signaling or macropinocytosis causes an increase in BMP signaling and NMJ overgrowth characterized by an excess of satellite boutons (Bogdan et al., 2004; Kim et al., 2019; Qurashi et al., 2007; Schenck et al., 2003; Schenck et al., 2004; Zhao et al., 2013).

Rac1-SCAR signaling has also been implicated in other synaptic processes. For example, a genetic study in *Caenorhabditis elegans* showed that a signaling pathway consisting of CED-5 (a Rac guanine nucleotide exchange factor [GEF]), CED-10/Rac1, and MIG-10/Lamellipodin acts downstream of the Netrin receptor UNC-40 to instruct synaptic vesicle (SV) clustering during neurodevelopment (Stavoe and Colón-Ramos, 2012). Subsequently, MIG-10 was shown to interact with the *C. elegans* homolog of Abi (ABI-1) to instruct SV clustering (Stavoe et al., 2012). Despite these findings, nothing is known about whether actin polymerization by Rac1-SCAR signaling is also involved in SV regulation and other cellular processes at mature synapses.

At the *Drosophila* NMJ, SVs are subdivided into at least two functionally distinct pools: the active cycling pool (also called the exo/endo cycling pool, ECP) and the reserve pool (RP; Delgado et al., 2000; Kuromi and Kidokoro, 1998; Kuromi and Kidokoro, 2002; Rizzoli and Betz, 2005). The ECP maintains synaptic transmission during low-frequency (≤3 Hz) or high K^+^ stimulation, while the RP is efficiently recruited for release only during high-frequency stimulation (≥10 Hz; Delgado et al., 2000; Kuromi and Kidokoro, 2000; Kuromi and Kidokoro, 2002; Verstreken et al., 2005). The formation and dynamics of RP vesicles at the *Drosophila* NMJ critically depend on actin polymerization (Delgado et al., 2000; Kuromi and Kidokoro, 1998; Kuromi and Kidokoro, 1999) and are required for sustaining neurotransmitter release during prolonged high-frequency stimulation and expression of post-tetanic potentiation (PTP), a form of short-term plasticity (Kim et al., 2009; Verstreken et al., 2005). However, little is known about actin-regulatory pathways required for proper regulation of vesicle dynamics.

Here, we identify and characterize a core actin-regulatory pathway required for normal regulation of synaptic growth and tetanic stimulation-induced short-term plasticity. We provide evidence that *Drosophila* Vav, a Rho-type GEF, acts upstream of the Rac1-SCAR signaling pathway to restrain synaptic growth by mediating macropinocytosis, which is associated with BMPR degradation and signal attenuation (Kim et al., 2019). We then provide evidence that the Vav-Rac1-SCAR cascade facilitates tetanus-induced changes in presynaptic release by mediating RP mobilization. Finally, we show that the roles of Vav in macropinocytosis and RP mobilization are genetically separable. This study uncovers a novel upstream regulator of presynaptic actin dynamics that independently modulates structural and functional presynaptic plasticity through two distinct cellular mechanisms.

## Results

### Presynaptic Vav is required for normal NMJ growth

To identify new genes controlling synaptic development, we screened 1,500 P-element transposon mutants based on immunohistochemical inspection of the *Drosophila* NMJ using the anti-HRP neuronal membrane marker (Nahm et al., 2010). This screen allowed us to identify a P-element insertion (*Vav*^*KG02022*^) localized within the *Vav* gene (*CG7893*). NMJs in *Vav*^*KG02022*^ mutants were more extensive than in WT (*w*^*1118*^) controls (data not shown).

**Figure 1.**
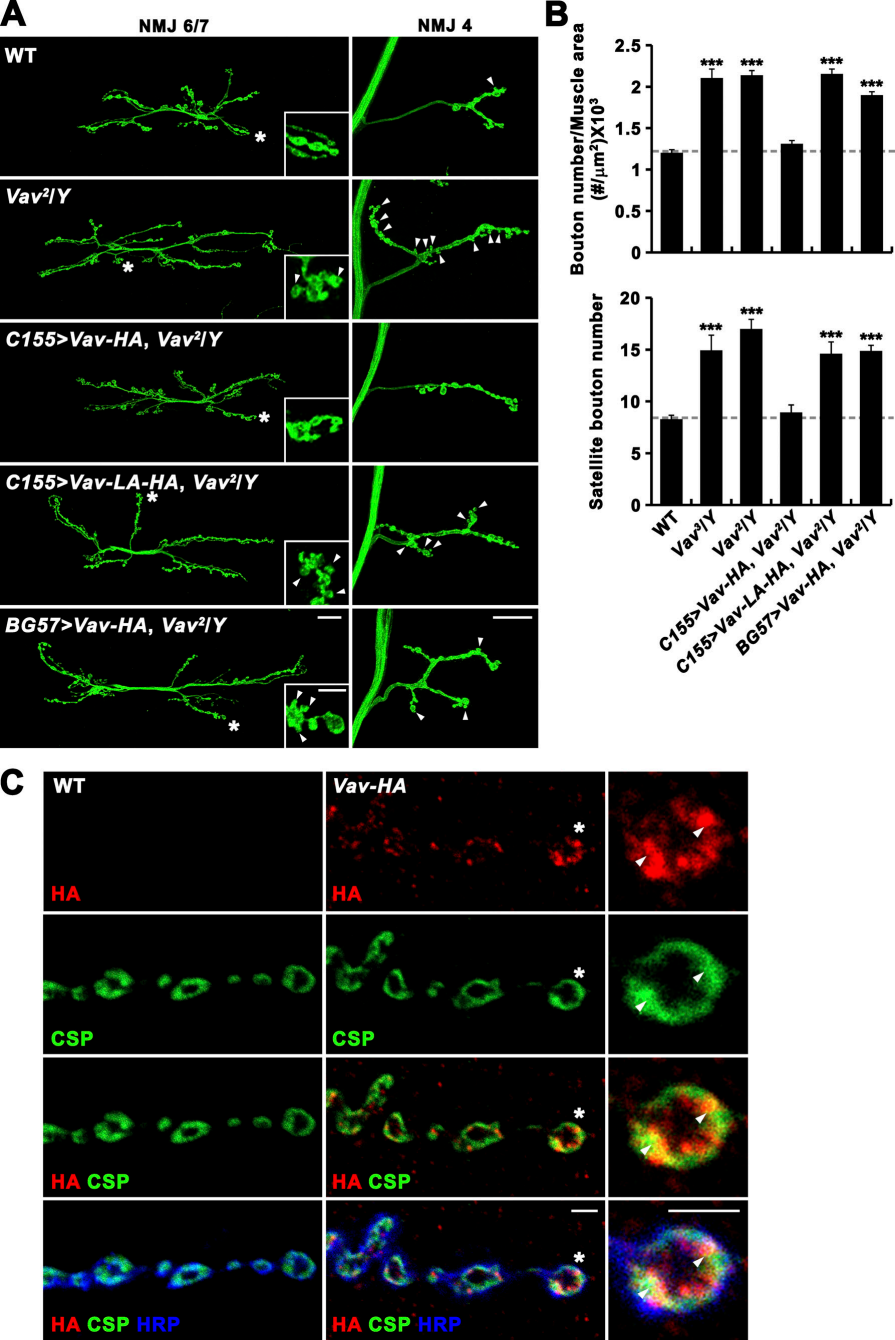
**Function and expression of Vav at the larval NMJ. (A and B)** Vav controls synaptic growth. **(A)** Confocal images of anti-HRP–labeled NMJs 6/7 and 4 in WT (*w*^*1118*^), *Vav*^*2*^/*Y*, *Vav*^*2*^,*C155-GAL4*/*Y*; *UAS-Vav-HA*/+ (*C155>Vav-HA*, *Vav*^*2*^*/Y*), *Vav*^*2*^,*C155-GAL4*/*Y*; *UAS-Vav-L443A-HA*/+ (*C155>Vav-LA-HA*, *Vav*^*2*^*/Y*), and *Vav*^*2*^/*Y*; *BG57-GAL4*/*UAS-Vav-HA* (*BG57>Vav-HA*, *Vav*^*2*^*/Y*) third instar larvae. Insets show higher magnification views of terminal boutons marked by asterisks. Arrowheads indicate satellite boutons. **(B)** Quantification of total bouton number normalized to muscle surface area and satellite bouton number at NMJ 6/7 in the indicated genotypes (*n* = 15). Data represent mean ± SEM. Statistically significant differences versus WT are indicated (***, P < 0.001). Dashed lines represent mean WT values. **(C)** Vav localization at the NMJ. Confocal images of NMJs 6/7 labeled with anti-HA, anti-Cysteine-string protein (CSP), and anti-HRP antibodies are shown for WT and CRISPR Vav-HA line. Right panels show higher magnification views of a single bouton (asterisk) in CRISPR Vav-HA line. Arrowheads indicate intracellular spot structures that are enriched with Vav-HA and CSP. Scale bars: 20 μm (A); 5 μm (A, inset); 2 μm (C).

**Figure S1.**
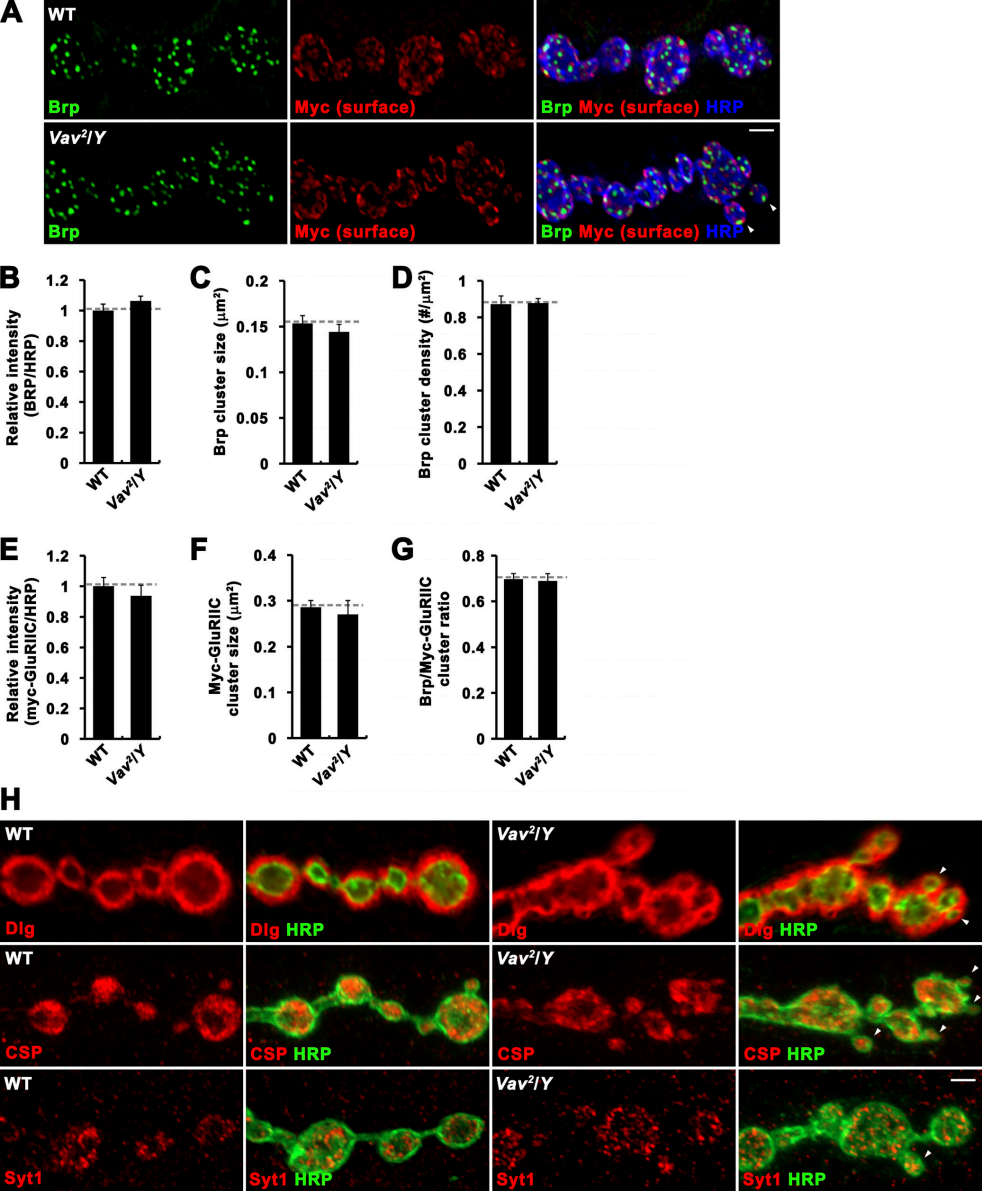
**Anatomical features of *Vav* mutant NMJs are grossly normal. (A)** Single confocal slices of NMJ 6/7 in *BG57-GAL4/UAS-Myc-GluRIIC-Flag* (WT) and *Vav*^*2*^*/Y*; *BG57-GAL4/UAS-Myc-GluRIIC-Flag* (*Vav*^*2*^*/Y*) third instar larvae. Larval fillets were triply stained with anti-Myc (red) under nonpermeant conditions and then following permeabilization with anti-Brp (green) and anti-HRP (blue). **(B–G)** Quantification of Brp/HRP intensity ratio (B), Brp cluster size (C), Brp cluster density (D), Myc-GluRIIC/HRP intensity ratio (E), Myc-GluRIIC cluster size (F), Brp/Myc-GluRIIC cluster number ratio (G). Data represent mean ± SEM. *n* = 75 NMJ branches from 15 larvae. Dashed lines represent mean WT values. **(H)** Confocal images of NMJ 6/7 doubly labeled with anti-HRP and anti-Dlg (top), anti-CSP (middle), or anti-Syt1 (bottom) are shown for WT and *Vav*^*2*^/*Y* third instar larvae. Satellite boutons are marked by arrowheads. Scale bars: 2 μm.

To further address the synaptic role of *Vav*, we utilized two previously reported null alleles, *Vav*^*2*^ and *Vav*^*3*^, that were generated by imprecise excision of *Vav*^*KG02022*^ (Malartre et al., 2010). Both *Vav*^*2*^ and *Vav*^*3*^ mutants died as pharate adults before eclosion, as previously described (Malartre et al., 2010). Like *Vav*^*KG02022*^ mutants, male third instar larvae hemizygous for *Vav*^*2*^ revealed NMJ overgrowth with an excess of immature satellite boutons at every type I NMJ, including NMJs 6/7 and 4 (Fig. 1 A). Bouton number at NMJ 6/7 in hemizygous *Vav*^*2*^ larvae was increased by 75% compared with WT controls (88.6 ± 2.9; *Vav*^*2*^/*Y*: 154.8 ± 6.0; mean ± SEM; P < 0.001). With normalization to muscle surface area, bouton number remained 78% larger in hemizygous *Vav*^*2*^ larvae compared with controls (WT: 1.20 ± 0.04 × 10^−3^ boutons/μm^2^; *Vav*^*2*^/*Y*: 2.14 ± 0.06 × 10^−3^ boutons/μm^2^; P < 0.001; Fig. 1 B). The number of satellite boutons per NMJ 6/7 was increased by 105% (WT: 8.3 ± 0.4; *Vav*^*2*^/*Y*: 17.0 ± 0.9; P < 0.001; Fig. 1 B). Comparable defects in synaptic growth were observed in hemizygous *Vav*^*3*^ larvae (Fig. 1 B). Despite synaptic overgrowth, *Vav*^*2*^ mutants had no gross defects in the levels or distribution of several synaptic markers, including active zone protein Bruchpilot, SV proteins (Cysteine-string protein [CSP] and Synaptotagmin 1 [Syt1]), glutamate receptor subunit GluRIIC, and subsynaptic reticulum marker discs-large (Fig. S1, A–H).

To determine whether *Vav* is required pre- or post-synaptically for normal synaptic growth, we expressed the Vav isoform C with a C-terminal HA tag (Vav-HA) in *Vav*^*2*^ mutants using the UAS/GAL4 system (Brand and Perrimon, 1993). Expression of *UAS-Vav-HA* transgene in all postmitotic neurons using *C155-GAL4* completely rescued the phenotypes of increased bouton number and satellite bouton number in *Vav*^*2*^ hemizygotes (Fig. 1, A and B). By contrast, expression of *UAS-Vav-HA* in somatic muscles using *BG57-GAL4* failed to rescue the synaptic overgrowth (Fig. 1, A and B), indicating presynaptic requirement for Vav. To investigate the underlying mechanism, we tested the effect of the point mutation L443A in Vav (Vav-L443A) on rescue activity. An analogous mutation in mammalian Vav abolishes its GEF activity (Crespo et al., 1997). Neuronal expression of *UAS-Vav-L443A-HA* failed to rescue the synaptic overgrowth phenotype in *Vav*^*2*^ hemizygotes (Fig. 1, A and B). These findings imply that Vav acts pre-synaptically through the GEF domain to regulate synaptic growth at the NMJ.

To visualize Vav localization within the presynaptic nerve terminal at the NMJ, we employed CRISPR/Cas9-based genome engineering to insert an HA tag into the endogenous *Vav* locus (*Vav-HA*) and used anti-HA to detect Vav-HA protein. Vav-HA largely localized to punctate or spot structures that were distributed throughout the presynaptic nerve terminal and the muscle cytoplasm (Fig. 1 C). In the presynaptic nerve terminal, Vav-HA puncta highly overlapped with CSP, with some portion associated with the presynaptic membrane (Fig. 1 C).

### Vav restricts synaptic growth via inhibition of BMP signaling

**Figure 2.**
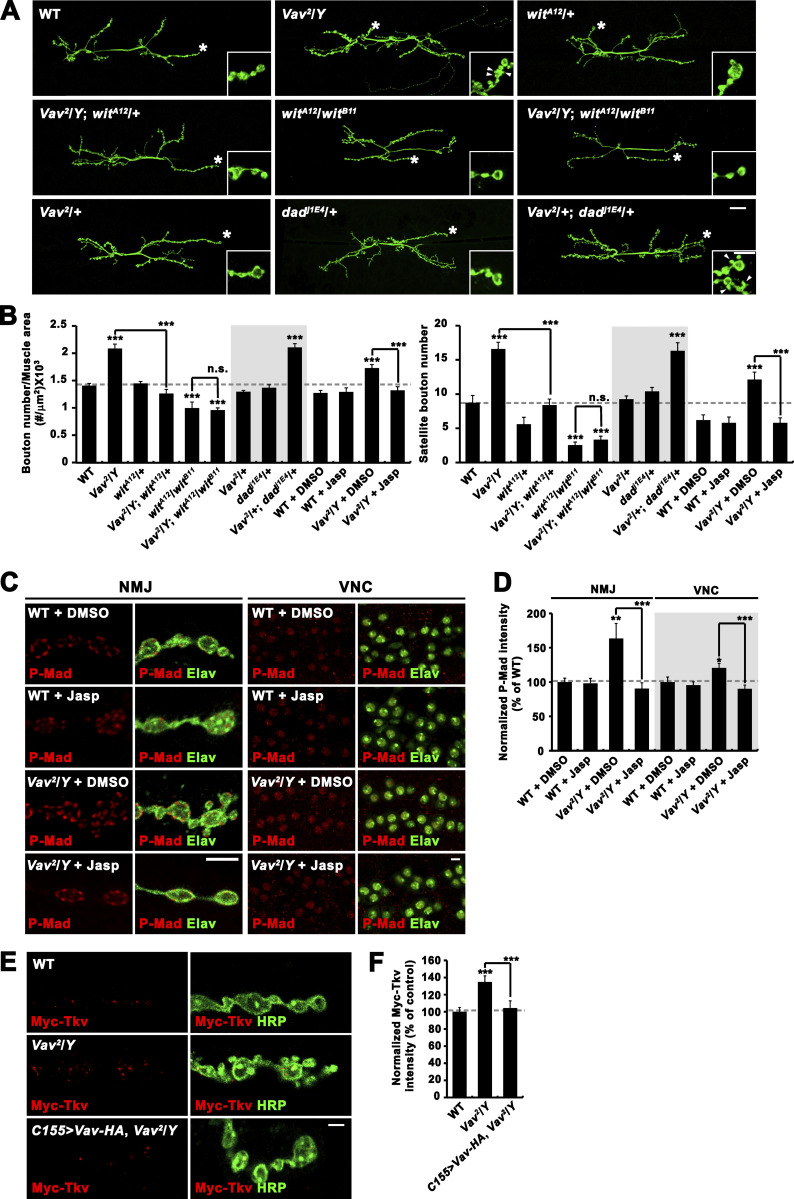
**Vav restrains synaptic growth by downregulating BMP signaling. (A and B)** Genetic interactions between *Vav* and BMP pathway components. **(A)** Confocal images of anti-HRP–labeled NMJ 6/7 in third instar larvae of indicated genotypes. Insets show magnified views of terminal boutons marked with asterisks. **(B)** Quantification of total bouton number normalized to muscle area and satellite bouton number at NMJ 6/7 (*n* = 15). In some experiments, WT and Vav^2^/*Y* mutant larvae were grown on medium containing 0.1% DMSO (vehicle alone) or 10 μM jasplakinolide. **(C and D)** Loss of *Vav* increases levels of P-Mad in motor neurons. **(C)** Single confocal sections of NMJ 6/7 and VNC labeled with anti–P-Mad and anti-HRP (NMJ) or anti-Elav (VNC) in WT and *Vav*^*2*^/*Y* third instar larvae grown on medium containing 0.1% DMSO or 10 μM jasplakinolide. **(D)** Quantification of P-Mad intensity normalized to HRP (NMJ) or Elav (VNC). *n* = 18 NMJs or VNCs. **(E and F)** Loss of *Vav* increases Myc-Tkv levels at the NMJ. **(E)** Single confocal sections of NMJ 6/7 doubly labeled with anti-Myc (red) and anti-HRP (green) shown for *C155-GAL4*/*Y*; *UAS-Myc-tkv*/+ (WT), *Vav*^*2*^,*C155-GAL4*/*Y*; *UAS-Myc-tkv*/+ (*Vav*^*2*^/*Y*), and *Vav*^*2*^,*C155-GAL4*/*Y*; *UAS-Myc-tkv*,*UAS-Vav-HA*/+ (*C155>Vav-HA*, *Vav*^*2*^*/Y*) third instar larvae. **(F)** Quantification of the ratio of Myc-Tkv to HRP intensities. *n* = 18 NMJs. Data represent mean ± SEM. Comparisons are with WT (***, P < 0.001; n.s., not significant). Dashed lines represent mean WT or WT+DMSO values. Scale bars: 20 μm (A); 5 μm (A, inset); 5 μm (C); 2 μm (E).

In *Drosophila*, elevation of retrograde BMP signaling causes NMJ overgrowth with excess satellite boutons (Nahm et al., 2013; O’Connor-Giles et al., 2008; Sweeney and Davis, 2002; Wang et al., 2007), recapitulating the *Vav* phenotype. To test whether synaptic overgrowth in *Vav* mutants might be due to elevated BMP signaling, we first examined genetic interaction between *Vav* and the BMP type II receptor *wit*. Heterozygosity for the *wit* null allele (*wit*^*A12*^) had no effect on NMJ morphology but fully suppressed synaptic overgrowth in *Vav*^*2*^ mutants (Fig. 2, A and B). Furthermore, *Vav*, *wit* double mutant (*Vav*^*2*^*/Y*; *wit*^*A12*^*/wit*^*B11*^) NMJs were severely undergrown, recapitulating the NMJ phenotype of *wit* single mutants (*wit*^*A12*^*/wit*^*B11*^; Fig. 2, A and B). These dosage-sensitive genetic interactions imply that synaptic overgrowth in *Vav* requires BMP signaling. We also examined genetic interaction between *Vav* and *dad*, which encodes an inhibitory Smad blocking BMP signaling. Each mutation in a heterozygous condition (*Vav*^*2*^/+ or *dad*^*j1E4*^/+) had no or mild effect on overall and satellite bouton numbers. However, the trans-heterozygosity for both mutations (*Vav*^*2*^/+; *dad*^*j1E4*^/+) strongly increased overall and satellite bouton numbers to levels comparable to those observed for hemizygous *Vav*^*2*^ mutants (Fig. 2, A and B), demonstrating a role for *Vav* in inhibiting BMP signaling during synaptic growth.

Next, we tested the impact of Vav loss on the accumulation of P-Mad at presynaptic terminals and in nuclei of motor neurons, as molecular readouts of BMP signaling activity (Marques et al., 2002; McCabe et al., 2003). P-Mad levels at both locations were significantly higher in *Vav*^*2*^ mutants relative to WT controls (P < 0.001; Fig. 2, C and D), confirming a role for Vav in downregulating BMP signaling activity in motor neurons. Combined with genetic interactions between *Vav* and BMP signaling pathway components, this result supports a model in which *Vav* restrains synaptic structural growth at the NMJ by inhibiting presynaptic BMP signaling.

### Vav acts upstream of Rac1 downregulating BMPRs through macropinocytosis

**Figure S2.**
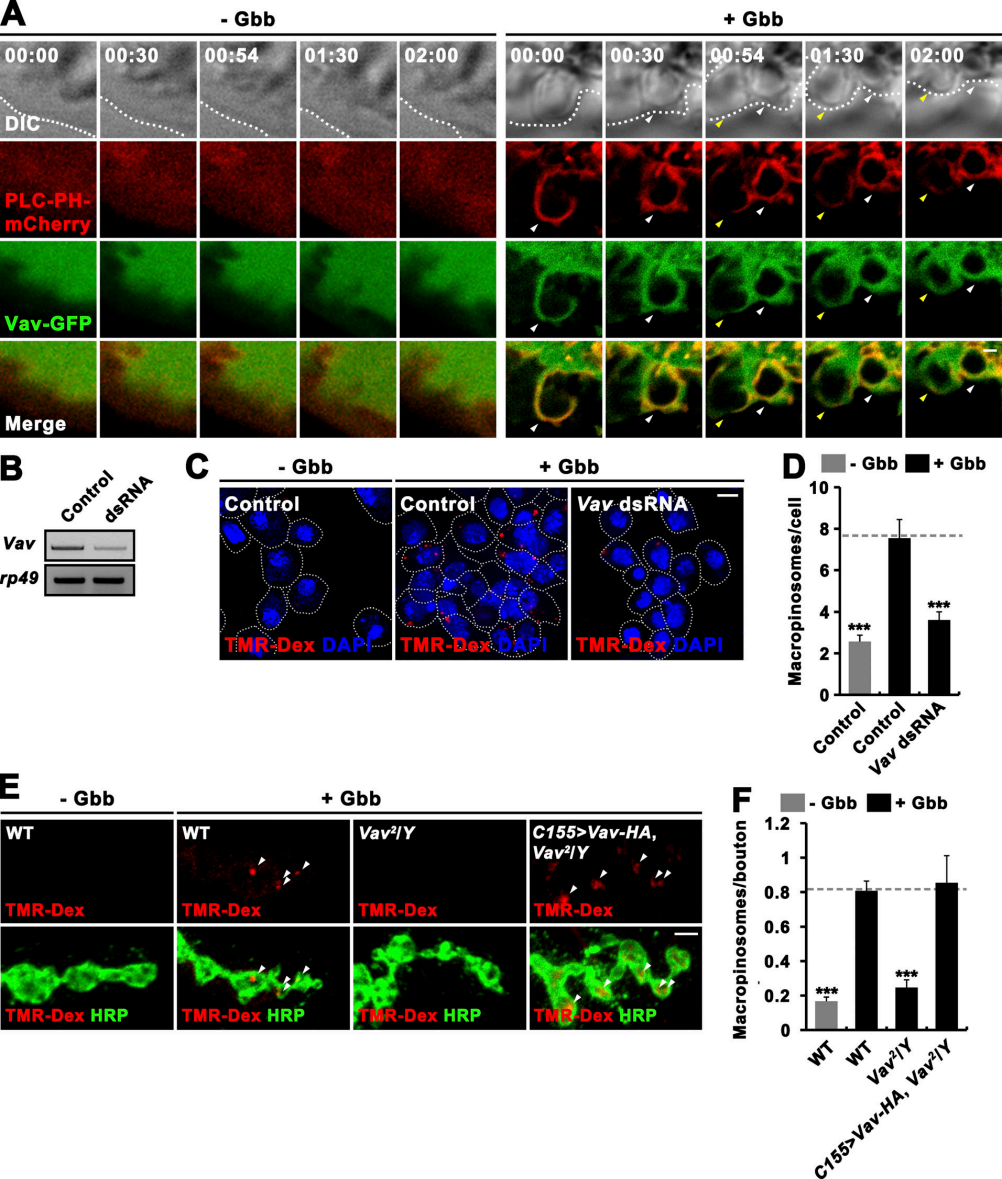
**Vav is required for Gbb-induced macropinocytosis. (A)** Time-lapse imaging of mock-treated (− Gbb) or Gbb-stimulated (+ Gbb) BG2-c2 cells expressing the PI(4,5)P_2_ probe PLC-PH-mCherry (red) and Vav-GFP (green). Differential interference contrast images are shown with elapsed times (min:s) in top panels. Note that in Gbb-stimulated cells, Vav-GFP is associated with PLC-PH-mCherry–labeled macropinocytic structures including ruffles, macropinocytic cups, and newly formed macropinosomes (arrowheads). **(B–D)** RNAi knockdown of *Vav* expression significantly impairs Gbb-induced macropinocytosis in BG2-c2 cells. **(B)** RT-PCR analysis of *Vav* and *rp49* RNA expression in mock-treated (control) and *Vav* dsRNA-transfected BG2-c2 cells. **(C)** Confocal images of control or *Vav*-knockdown BG2-c2 cells. Cells were incubated for 5 min with 2 mg/ml TMR-Dex (70 kD, red) in the absence or presence of 50 ng/ml recombinant Gbb and stained with DAPI (blue). **(D)** Quantification of the number of TMR-Dex-filled macropinosomes (puncta >0.2 μm in diameter) per cell. *n* = 60 cells. **(E and F)** Vav is required for Gbb-induced synaptic macropinocytosis. **(E)** Confocal images of NMJ 6/7 terminals stained with anti-HRP (green) following 5-min pulse of 2 mg/ml TMR-Dex (red) in the absence or presence of 50 ng/ml Gbb are shown for WT, *Vav*^*2*^/*Y*, and *C155-GAL4*/*Y*; *UAS-Vav-HA*/+ (*C155>Vav-HA*, *Vav*^*2*^*/Y*) larvae. Arrowheads indicate TMR-Dex–positive puncta. **(F)** Quantification of the number of TMR-Dex–positive puncta per bouton. *n* = 30 NMJ branches. Data represent mean ± SEM. ***, P < 0.001. Dashed lines represent mean Gbb-treated control or Gbb-treated WT values. Scale bars: 0.5 μm (A); 5 μm (C); 2 μm (E). Source data are available for this figure: SourceData FS2.

Vav has been characterized as a GEF for the small GTPase Rac1 (Couceiro et al., 2005; Hornstein et al., 2003). In addition, actin-regulatory Rac1-SCAR signaling inhibits synaptic growth by mediating Gbb-induced macropinocytosis, which is coupled with BMPR degradation and signal attenuation (Kim et al., 2019). We, therefore, hypothesized that Vav might regulate synaptic growth through the Rac1-SCAR pathway. To test this hypothesis, we first investigated whether Vav plays an essential role in Gbb-induced macropinocytosis. To this end, we examined the recruitment of Vav to macropinocytic structures in BG2-c2 neuronal cells expressing phospholipase Cδ1-pleckstrin homology domain-mCherry (PLC-PH-mCherry), a PIP_2_ reporter labeling early macropinocytic structures (Araki et al., 2007). Time-lapse, live-cell imaging showed Gbb-induced formation of PLC-PH-mCherry–positive membrane ruffles and macropinosomes (Fig. S2 A). Notably, these macropinocytic structures were found to recruit coexpressed Vav-GFP, suggesting a potential role of Vav in Gbb-induced macropinocytosis. We also analyzed the effect of Vav loss on Gbb-induced macropinocytosis. As previously reported (Kim et al., 2019), treatment with Gbb (50 ng/ml) potently induced the formation of tetramethylrhodamine-dextran (TMR-Dex)–positive macropinosomes in BG2-c2 cells and at larval NMJs (Fig. S2, B–F). Importantly, this Gbb-induced TMR-Dex uptake was abrogated in Vav-depleted BG2-c2 cells or at *Vav*^*2*^ mutant NMJs (Fig. S2, B–F). Expression of *UAS-Vav-HA* in *Vav*^*2*^ mutants using the *C155-GAL4* driver fully restored Gbb-induced macropinocytosis (Fig. S2, E and F), demonstrating an essential role for Vav in Gbb-induced presynaptic macropinocytosis.

We then investigated the impact of Vav loss on steady-state synaptic levels of neuronally expressed Myc-tagged Tkv (Myc-Tkv) at the NMJ. Myc-Tkv levels were increased by ∼31% in hemizygous *Vav*^*2*^ mutants compared with control larvae (Fig. 2, E and F). This phenotype was completely rescued by expressing *UAS-Vav-HA* in neurons using the *C155-GAL4* driver, demonstrating a role for Vav in downregulating synaptic BMPRs.

**Figure 3.**
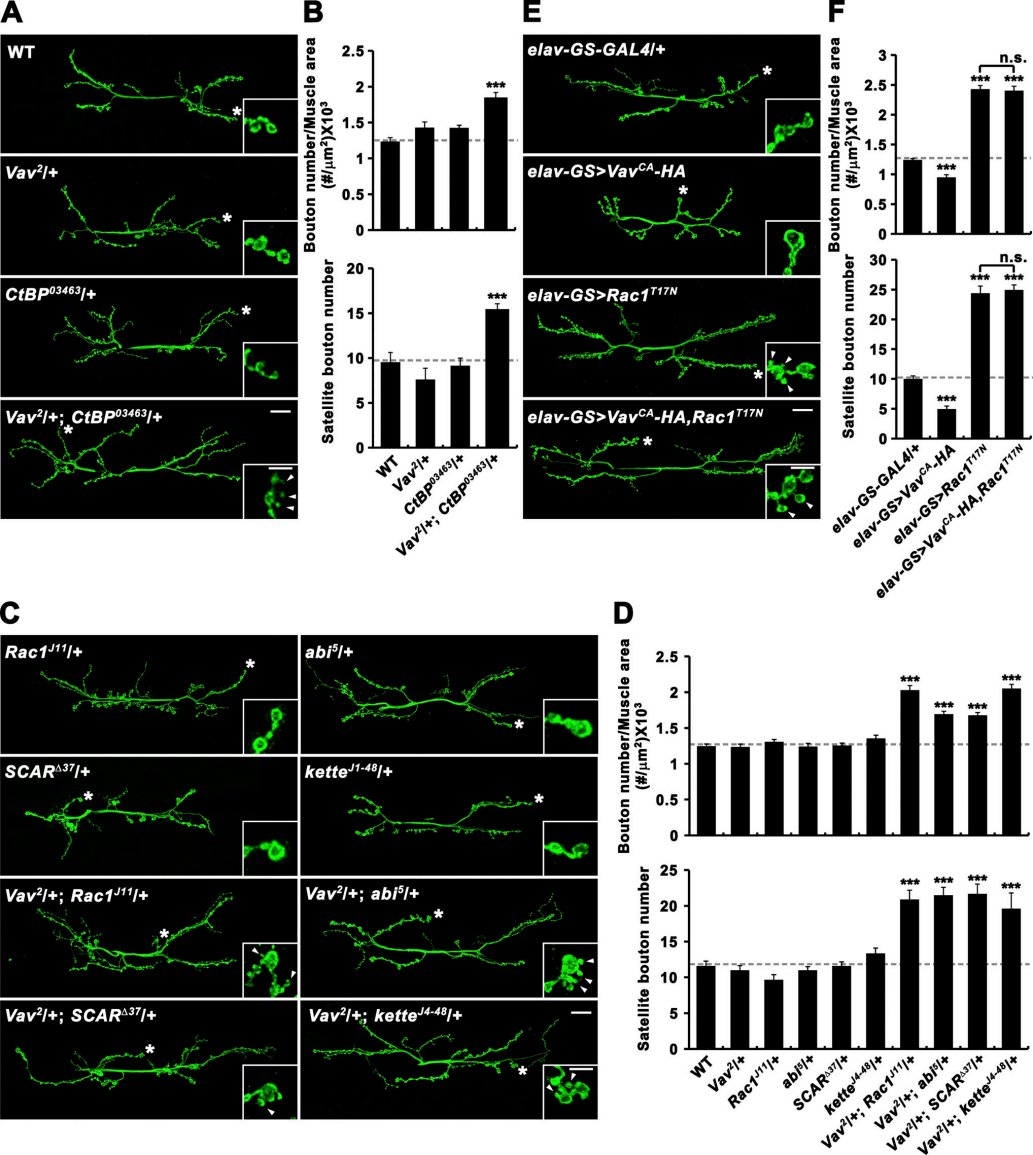
***Vav* functionally interacts with regulators of macropinocytosis and components of the Rac1-SCAR pathway during synaptic growth. (A and B)** Trans-heterozygous genetic interaction between *Vav* and *CtBP* during synaptic growth. **(A)** Confocal images of anti-HRP–labeled NMJ 6/7 in WT, *Vav*^*2*^/+, *CtBP*^*03463*^/+, and *Vav*^*2*^/+; *CtBP*^*03463*^/+ third instar larvae. **(B)** Quantification of total bouton number normalized to muscle area and satellite bouton number. *n* = 15 NMJs. **(C and D)** Trans-heterozygous genetic interactions between *Vav* and Rac1-SCAR pathway components. **(C)** Confocal images of anti-HRP–labeled NMJ 6/7 in third instar larvae of indicated genotypes. **(D)** Quantification of total bouton number normalized to muscle area and satellite bouton number. *n* = 15 NMJs. **(E and F)** Epistatic analysis of the relationship between *Vav* and *Rac1*. **(E)** Confocal images of anti-HRP–labeled NMJ 6/7 in *elav-GS-GAL4*/+, *UAS-Vav*^*CA*^*-HA*/+; *elav-GS-GAL4*/+ (*elav-GS>Vav*^*CA*^*-HA*), *elav-GS-GAL4*/*UAS-Rac1*^*T17N*^ (*elav-GS>Rac1*^*T17N*^), and *UAS-Vav*^*CA*^*-HA*/+; *elav-GS-GAL4*/*UAS-Rac1*^*T17N*^ (*elav-GS>Vav*^*CA*^*-HA,Rac1*^*T17N*^) third instar larvae. Animals were fed with RU486 during larval development. **(F)** Quantification of total bouton number normalized to muscle area and satellite bouton number. *n* = 18 NMJs. Insets show magnified views of terminal boutons marked with asterisks. Data represent mean ± SEM. Comparisons are with WT (***, P < 0.001; n.s., not significant). Dashed lines represent mean WT or *elav-GS-GAL4*/+ values. Scale bars: 20 μm; 5 μm (insets).

We also examined trans-heterozygous genetic interaction between *Vav* and *C-terminal binding protein* (*CtBP*), a key regulator of macropinocytosis, during synaptic growth. Total and satellite bouton numbers were significantly increased by removing one copy each of *Vav* and *CtBP* (*Vav*^*2*^/+; *CtBP*^*0346*3^/+), whereas loss of one copy of either had no effect (Fig. 3, A and B), supporting a model wherein Vav regulates synaptic growth via macropinocytosis.

Next, we examined genetic interactions between *Vav* and Rac1-SCAR pathway components during synaptic growth. In contrast to normal synaptic growth in individual heterozygotes, we observed strong synaptic overgrowth in larvae trans-heterozygous for *Vav*^*2*^ and *Rac1*^*J11*^, *abi*^*5*^, *SCAR*^*Δ37*^, or *kette*^*J4-48*^ (Fig. 3, C and D), supporting a functional link between Vav and the Rac1-SCAR pathway. We next pursued genetic epistasis experiment to further examine the functional relationship between *Vav* and *Rac1*. Neuronal overexpression of constitutively active Vav (Vav^CA^) caused synaptic undergrowth, while neuronal overexpression of dominant negative Rac1 (Rac1^T17N^) had the opposite effect (Fig. 3, E and F). Importantly, overall and satellite bouton numbers at NMJ 6/7 in larvae co-overexpressing Vav^CA^ and Rac1^T17N^ were essentially the same as in larvae expressing Rac1^T17N^ alone (Fig. 3, E and F), placing Vav upstream of Rac1 in the same pathway controlling synaptic growth.

Lastly, we tested whether the synaptic overgrowth phenotype of *Vav* mutants can be pharmacologically rescued by feeding larvae with jasplakinolide (10 μM), an actin filament (F-actin) polymerizing and stabilizing drug (Bubb et al., 1994). Jasplakinolide completely rescued the synaptic overgrowth and increased P-Mad phenotypes in *Vav*^*2*^ mutants, with no effect on WT (Fig. 2, B–D), suggesting that Vav inhibits BMP signaling through modulation of synaptic actin dynamics.

Altogether, our findings support the model that Vav limits BMP-mediated synaptic growth via activation of the actin-regulatory Rac1-SCAR pathway mediating macropinocytosis.

### *Vav* mutants display normal evoked release and synaptic ultrastructure

**Figure S3.**
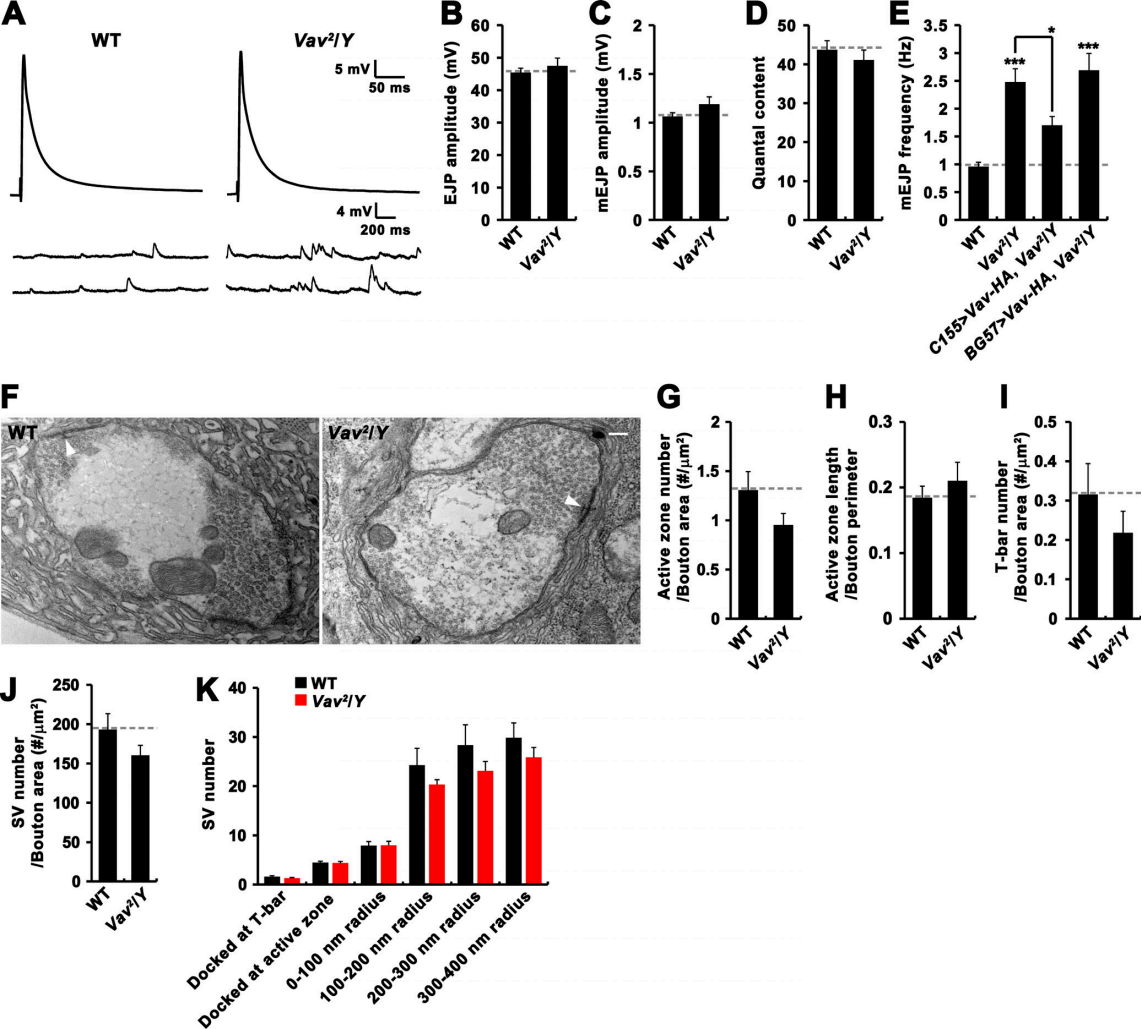
***Vav* mutants show normal evoked neurotransmission and presynaptic ultrastructure. (A)** Representative traces of EJPs and spontaneous mEJPs recorded from WT and *Vav*^*2*^/*Y* mutant NMJs (1.5 mM Ca^2+^). EJPs show an average of 20 consecutive recordings during nerve stimulation at 0.5 Hz. **(B–E)** Quantifications of mean EJP amplitude (B), mEJP amplitude (C), quantal content (D), and mEJP frequency (E) in WT, *Vav*^*2*^/*Y*, *Vav*^*2*^,*C155-GAL4*/*Y*; *UAS-Vav-HA*/+ (*C155>Vav-HA*, *Vav*^*2*^/*Y*), and *Vav*^*2*^/*Y*; *BG57-GAL4*/*UAS-Vav-HA* (*BG57>Vav-HA*, *Vav*^*2*^/*Y*). *n* = 15 larvae. **(F)** Representative TEM images of WT and *Vav*^*2*^/*Y* mutant NMJ boutons (type Ib). The mutant has normal bouton size, morphology, and postsynaptic subsynaptic reticulum. White arrowheads indicate T-bars. **(G–K)** Quantification of ultrastructural phenotypes, including active zone number (G), active zone length (H), T-bar number (I), SV density (J), and the number of SVs either docked or located in an area of <100, 100–200, 200–300, and 300–400 nm around the active zone T-bar. *n* = 21 boutons. Data represent mean ± SEM. Comparisons are with WT (*, P < 0.05; ***, P < 0.001). Dashed lines represent mean WT values. Scale bar: 200 nm.

To assess the effect of Vav loss on synaptic function, we performed intracellular recordings from muscle 6 of third instar larvae. We stimulated the motor nerve at a low frequency (0.5 Hz) in the presence of 1.5 mM external Ca^2+^. The mean amplitudes of excitatory junctional potentials (EJPs) or spontaneous miniature EJPs (mEJPs) were not significantly altered in hemizygous *Vav*^*2*^/*Y* mutants compared with WT controls (Fig. S3, A–C). Thus, quantal content (ratio of mean EJP amplitude to mean mEJP amplitude) remained unaltered in *Vav*^*2*^ mutants (Fig. S3 D). However, we found a significant increase in the frequency of miniature events (Fig. S3, A and E). This defect was strongly rescued by presynaptic, but not postsynaptic, expression of Vav-HA in *Vav*^*2*^ mutants (Fig. S3 E).

We next performed transmission electron microscopy (TEM) to assess ultrastructural features of *Vav* mutant synapses. The appearance of presynaptic boutons in *Vav*^*2*^/*Y* mutants was not significantly different from WT controls (Fig. S3 F). Likewise, the numbers of active zones and T-bars and the average length of active zones were not measurably altered by loss of *Vav* activity (Fig. S3, G–I). Furthermore, SV density and distribution were also normal in *Vav*^*2*^/*Y* mutants (Fig. S3, J and K). Thus, the ultrastructure of presynaptic boutons at the NMJ is not grossly affected by loss of Vav.

### Synaptic plasticity upon high-frequency stimulation is impaired in *Vav* mutants

**Figure 4.**
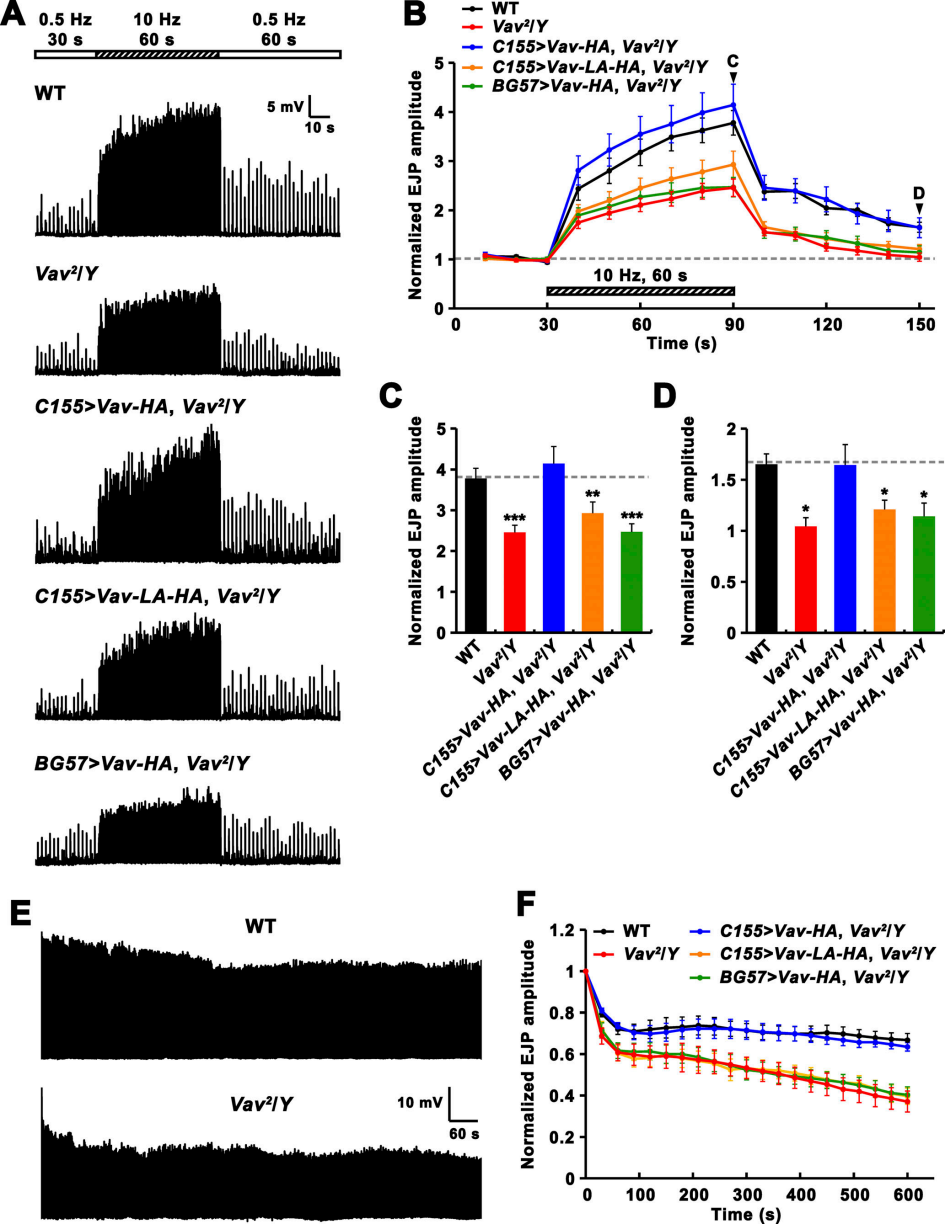
***Vav* mutants display alterations in activity-dependent synaptic plasticity. (A–D)** High-frequency stimulation under low Ca^2+^ reveals impaired synaptic augmentation and PTP in *Vav* mutants. **(A)** Representative recordings from WT, *Vav*^*2*^/*Y*, *Vav*^*2*^,*C155-GAL4*/*Y*; *UAS-Vav-HA*/+ (*C155>Vav-HA*, *Vav*^*2*^/*Y*), *Vav*^*2*^,*C155-GAL4*/*Y*; *UAS-Vav-L443A-HA*/+ (*C155>Vav-LA-HA*, *Vav*^*2*^/*Y*), and *Vav*^*2*^/*Y*; *BG57-GAL4*/*UAS-Vav-HA* (*BG57>Vav-HA*, *Vav*^*2*^/*Y*) larvae in 0.3 mM Ca^2+^. The stimulation paradigm was 0.5 Hz for 30 s (white bar), 10 Hz for 60 s (hatched bar), and 0.5 Hz for the remainder of experiment (white bar). **(B)** Plot of mean EJP amplitudes normalized to initial mean EJP amplitude (for the 0.5 Hz, 30 s control period) are shown over time for indicated genotypes. Each point in the ordinate represents the mean normalized amplitude for every 10 s. **(C and D)** Bar graphs of mean normalized EJP amplitudes at the end of (C) and at 60 s after (D) tetanic stimulation. *n* = 18 larvae. **(E and F)** High-frequency stimulation at a high Ca^2+^ concentration reveals enhanced synaptic depression in *Vav* mutants. **(E)** Representative recordings from WT and *Vav*^*2*^/*Y* larval NMJs in 10 mM Ca^2+^ saline during 10 Hz stimulation. **(F)** Plot of mean EJP amplitudes normalized to mean initial amplitude for indicated genotypes. Each point in the ordinate represents the mean normalized amplitude for every 30 s. *n* = 15 larvae. Data represent mean ± SEM. Comparisons are with WT (*, P < 0.05; **, P < 0.01; ***, P < 0.001). Dashed lines represent mean pre- or post-tetanus WT values.

The *Drosophila* NMJ shows robust augmentation of synaptic transmission and PTP in response to prolonged high-frequency stimulation (Rohrbough et al., 2000; Zhong and Wu, 1991). We asked whether these forms of short-term synaptic plasticity might be altered at *Vav*^*2*^ mutant NMJs under low Ca^2+^ (0.3 mM). The experimental paradigm consisted of initial nerve stimulation at 0.5 Hz for 30 s, followed by application of a tetanic stimulus train (10 Hz) for 60 s and basal stimulation at 0.5 Hz stimulation for 60 s (Fig. 4 A). During the tetanus train, WT NMJs showed rapid facilitation followed by gradual increase in EJP amplitude, leading to 3.8-fold augmentation relative to the initial mean amplitude of EJPs (Fig. 4, A–C). The amplitude of WT EJPs was potentiated 2.4-fold in the initial PTP phase (within 10 s after tetanic stimulation; Fig. 4, A and B). This early potentiation gradually declined with time, leading to PTP of ∼65% over basal EJP amplitude at 60 s after tetanic stimulation (Fig. 4, A, B, and D). By contrast, *Vav*^*2*^ mutants displayed severely reduced augmentation and impaired PTP. For instance, mutant EJPs showed only 2.5-fold potentiation at the end of the train and 1.6-fold potentiation in the initial PTP phase (Fig. 4, A–C). In addition, EJP amplitude at 60 s after tetanic stimulation did not significantly differ from basal EJP amplitude (Fig. 4 D). Presynaptic, but not postsynaptic, expression of *UAS-Vav-HA* in *Vav*^*2*^ mutants restored augmentation and PTP to WT levels (Fig. 4, A–D). However, presynaptic expression of *UAS-Vav-L443A-HA* did not rescue the same phenotypes (Fig. 4, A–D), demonstrating the importance of Vav GEF activity for tetanus-induced synaptic plasticity.

To further challenge *Vav* mutant synapses, we applied tetanic stimulation (10 Hz) for 10 min at a high external Ca^2+^ concentration (10 mM). In WT larvae, EJP amplitudes rapidly declined during the first 1 min of the train and then maintained at ∼67% of the initial values during the remainder of stimulation (Fig. 4, E and F). In hemizygous *Vav*^*2*^ mutants, however, EJPs displayed faster and greater depression during the initial 1-min period and then gradually decreased to <37% of initial amplitudes during the following period. This enhanced rundown phenotype was rescued by presynaptic, but not postsynaptic, expression of *UAS-Vav-HA*. Presynaptic expression of *UAS-Vav-L443A-HA* again failed to rescue the synaptic rundown phenotype (Fig. 4, E and F), indicating that Vav GEF activity is also required for maintaining normal synaptic transmission during tetanic stimulation.

### Mobilization of RP vesicles is disrupted in *Vav* mutants

**Figure S4.**
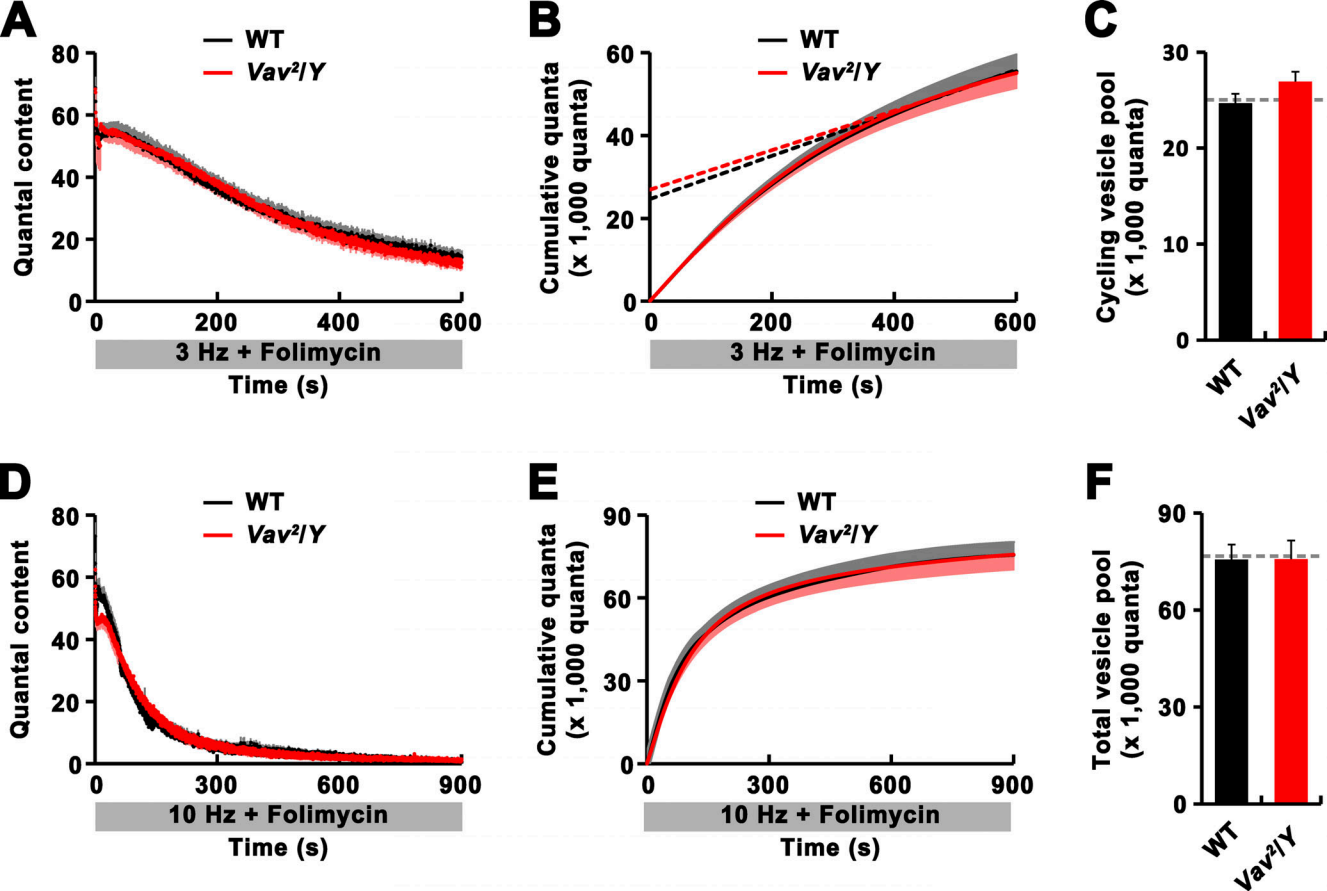
**The sizes of the ECP and the total vesicle pool are normal in *Vav* mutants. (A–C)** Analysis of ECP size. **(A)** Time course of synaptic depression at WT and *Vav*^*2*^/*Y* mutant NMJs during continuous 3 Hz stimulation in 2 mM Ca^2+^ saline with 1 μM folimycin. Martin correction factor was applied to correct for nonlinear summation during intracellular recordings. **(B)** Cumulative quantal plot of A. The estimate of ECP size was obtained from the y-intercept of a linear regression line back-extrapolated from points of cumulative quanta during the period between 400 and 600 s of continuous stimulation at 3 Hz. **(C)** Quantification of mean ECP sizes estimated in B. **(D–F)** Analysis of total vesicle pool size. **(D)** Time course of synaptic depression in WT and *Vav*^*2*^/*Y* larvae during continuous 10 Hz stimulation in 2 mM Ca^2+^ saline with 1 μM folimycin. **(E)** Cumulative quantal plot of D. **(F)** Quantification of the mean size of total vesicle pool as estimated by integrating quantal content over a 10 Hz, 900 s stimulation period. *n* = 8 larvae. Data represent mean ± SEM. Note that the sizes of ECP and total vesicle pool are not significantly different between WT and *Vav*^*2*^/*Y* larvae (P > 0.05). Dashed lines represent mean WT values.

At the third instar NMJ, defects in the cycling or maintenance of ECP and RP vesicles can affect synaptic strength in response to tetanic stimulation (Acharya et al., 2006; Geng et al., 2016; Kim et al., 2009; Kuromi and Kidokoro, 2000; Verstreken et al., 2005). To decipher the cellular mechanism underlying alterations in tetanus-induced plasticity in *Vav* mutants, we investigated a role for Vav in regulating SV dynamics. We first used an electrophysiological approach to assess the sizes of the ECP and the total vesicle pool. To estimate ECP size, NMJ preparations were continuously stimulated at 3 Hz in the presence of 1 μM folimycin, which blocks the refilling of recycling vesicles with neurotransmitters (Sankaranarayanan and Ryan, 2001). Under these conditions, synaptic depression occurred with an initial rapid phase, which primarily reflects depletion of ECP vesicles, and a late slower phase, which represents slow mixing of RP and ECP vesicles (Kim et al., 2009). The depression kinetics of *Vav*^*2*^ mutants in the initial and late phases were almost identical to those of WT (Fig. S4 A). When linear regression was used for points from the late phase of depression in a cumulative quantal plot (Delgado et al., 2000; Kim et al., 2009), ECP estimates (y-intercepts) of WT and *Vav*^*2*^ motor terminals were similar (Fig. S4, B and C). We also estimated total vesicle pool size by depleting motor terminals of SVs at 10 Hz frequency in the presence of 1 μM folimycin (Fig. S4 D) and measuring cumulative quanta (Fig. S4, E and F). This electrophysiological estimate showed WT levels of total pool size in *Vav*^*2*^ mutants. Since the total vesicle content is the sum of ECP and RP vesicles, our data collectively indicate that RP size is also normal in *Vav* mutants.

**Figure 5.**
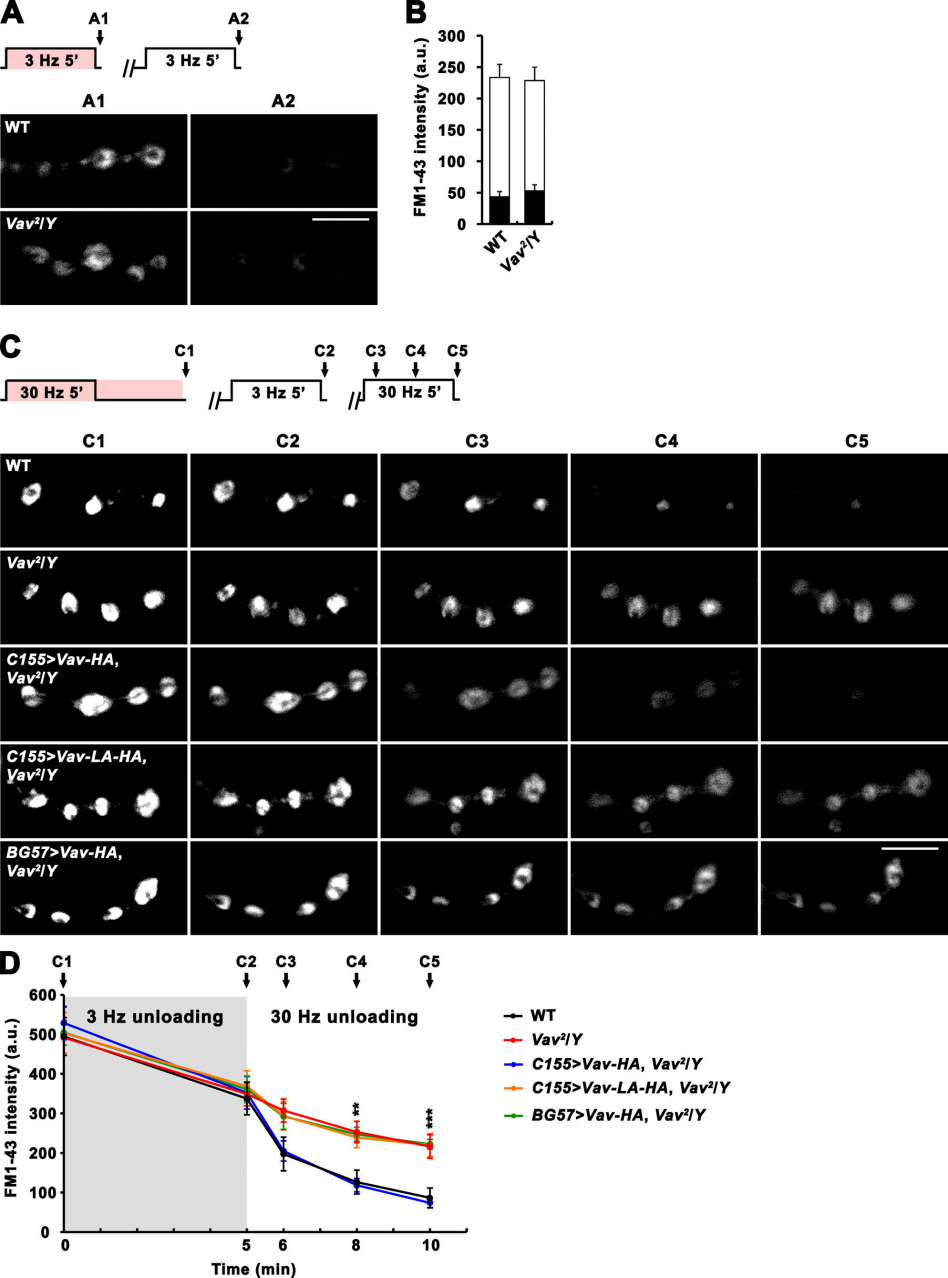
**Tetanus-induced RP mobilization is impaired in *Vav*. (A and B)** SV cycling through ECP is normal in *Vav* mutants. **(A)** Preparations from WT and *Vav*^*2*^/*Y* larvae were stimulated at 3 Hz for 5 min in 2 mM Ca^2+^ saline containing 4 μM FM 1–43 (pink color), washed with Ca^2+^-free saline, and imaged (A1; ECP loading). Loaded boutons were subsequently stimulated at 3 Hz for 5 min in 2 mM Ca^2+^ saline without dye and reimaged (A2; ECP unloading). **(B)** FM1-43 fluorescence intensity in loaded boutons before (height of whole columns) and after (height of black columns) 3 Hz unloading. **(C and D)** Tetanus-induced RP mobilization, but not RP formation, is impaired in *Vav* mutants. **(C)** Preparations from third instar larvae of indicated genotypes were stimulated at 30 Hz for 5 min in 2 mM Ca^2+^ saline containing 4 μM FM 1–43, incubated in the same bath for a further 5 min without stimulation, washed with Ca^2+^-free saline, and imaged (C1; ECP-RP loading). Loaded boutons were subsequently stimulated at 3 Hz for 5 min in 2 mM Ca^2+^ saline without dye and imaged (C2; ECP unloading). Finally, ECP-unloaded, RP-loaded boutons were repeatedly imaged after 1, 3, and 5 min of 30 Hz stimulation (C3–C5; RP unloading). **(D)** Fluorescence intensity in boutons is plotted against time after ECP-RP loading by 30 Hz stimulation (C1). Data represent mean ± SEM. Mean values were derived from 12 boutons from 6 different larvae. a.u., arbitrary units. Comparisons are with WT (**, P < 0.01; ***, P < 0.001). Scale bars: 5 μm.

Next, we employed the FM1-43 labeling technique to analyze endo- and exocytosis of ECP vesicles in *Vav* mutants. Loading of ECP vesicles with FM1-43 was achieved by stimulating the nerve at 3 Hz for 5 min in 2 mM Ca^2+^ saline-containing dye (Kuromi and Kidokoro, 2002). Under these conditions, WT and *Vav*^*2*^ mutant boutons internalized similar amounts of dye (Fig. 5, A and B). After ECP loading, the same synapses were re-stimulated at 3 Hz for 5 min in normal saline devoid of dye to mobilize loaded ECP vesicles. The remaining fluorescence after unloading was also similar in WT and *Vav*^*2*^ mutant boutons (Fig. 5, A and B). These results indicate that ECP vesicle dynamics during low-frequency stimulation are normal in *Vav* mutants and are consistent with the aforementioned conclusion that loss of Vav does not alter basal transmission.

Finally, we analyzed endo- and exocytosis of RP vesicles in *Vav* mutants using FM1-43 labeling. We first exposed NMJ boutons to 2 mM Ca^2+^ saline containing dye during (5 min, simultaneous load) and after (5 min, delayed load) nerve stimulation at 30 Hz. This protocol extensively loads both the ECP and RP with dye (Kuromi and Kidokoro, 2002). Under these conditions, WT and *Vav*^*2*^ mutant boutons displayed similar levels of FM1-43 fluorescence (Fig. 5, C1 and D), indicating that loading of the total vesicle pool occurs normally in *Vav* mutants. To visualize only loaded RP vesicles, we then unloaded the ECP by stimulating the same NMJs at 3 Hz for 5 min. Levels of the fluorescence that remained after the 3 Hz unloading were similar in both genotypes (Fig. 5, C2 and D), showing normal RP loading in *Vav* mutants. Finally, to investigate exocytosis of RP vesicles, we stimulated synapses at 30 Hz. The rate of dye unloading was strikingly reduced in *Vav*^*2*^ mutant boutons relative to WT controls (Fig. 5, C3–C5 and D). At the end of a 5-min tetanic stimulation, *Vav*^*2*^ mutants showed a significantly higher fluorescence (2.5 fold) compared with WT (Fig. 5 D). This RP mobilization defect was rescued by presynaptic expression of *UAS-Vav-HA* but not *UAS-Vav-L443A-HA* (Fig. 5 D). Thus, the GEF activity of presynaptic Vav is specifically required for RP mobilization during tetanic stimulation.

### Vav-mediated RP mobilization is required for PTP

**Figure 6.**
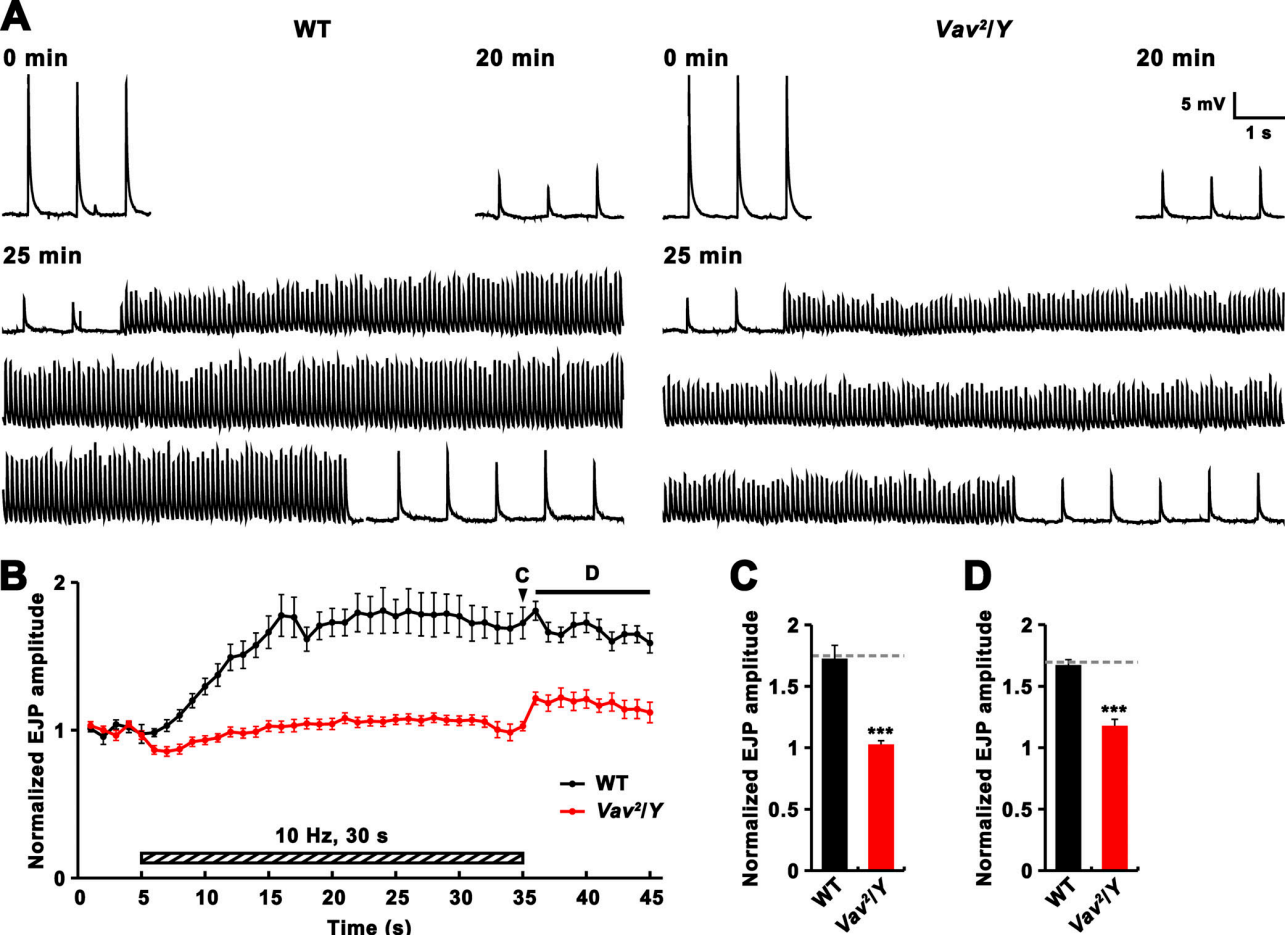
**Induction of PTP in WT and *Vav***^***2***^**/*Y* larval preparations depleted of ECP vesicles.** Larval preparations with severed axons were preincubated in 2 mM Ca^2+^ saline with 1 μM of the vesicle-refilling blocker folimycin for 5 min, and nerves were then stimulated at 1 Hz for 20 min to deplete ECP vesicles. This ECP-depletion step was followed by a resting period of 5 min without nerve stimulation and a 10 Hz stimulation for 30 s to mobilize RP vesicles, and terminated with a 1 Hz stimulation to assess the expression of PTP. **(A)** Representative EJP traces recorded from WT and *Vav*^*2*^/*Y* larval NMJs before, during, and after a 10 Hz, 30 s PTP induction protocol. **(B)** Plot of mean EJP amplitudes normalized to the mean amplitude of five consecutive EJPs just before the 10 Hz, 30 s PTP induction protocol. Each point in the ordinate represents mean normalized EJP amplitude for every 1 s. **(C and D)** Bar graphs of mean normalized EJP amplitudes for the last 1-s tetanus period (C) and for the first 10 s after cessation of tetanic stimulation (D). Data represent mean ± SEM. *n* = 12 larvae. Comparisons are with WT (***, P < 0.001). Dashed lines represent mean WT values.

Interfering with myosin light chain kinase (MLCK) blocks RP mobilization, disrupting PTP (Kim et al., 2009; Verstreken et al., 2005). We, therefore, asked if the reduced PTP in *Vav*^*2*^ mutants is attributable to the demonstrated defect in RP mobilization. To address this, we depleted ECP vesicles of glutamate by continuously stimulating the nerve at 1 Hz for 20 min in the presence of 1 μM folimycin. In WT and *Vav*^*2*^ mutant larvae, EJP amplitudes gradually declined during continuous 1 Hz stimulation and reached <20% of initial amplitude after 20 min of stimulation (Fig. 6 A). This reduction was not restored after a 5-min resting period, confirming ECP depletion (Fig. 6 A). A subsequent 10-Hz stimulation increased EJP amplitudes by 68% at WT NMJs, demonstrating recruitment of glutamate-filled RP vesicles. This enhanced transmission continued for more than 10 s after 10 Hz stimulation (Fig. 6, A–D), suggesting that the recruitment of RP vesicles continues even after tetanic stimulation. By contrast, *Vav*^*2*^ mutant NMJs displayed no obvious augmentation of EJP amplitude during 10 Hz stimulation (Fig. 6, A–D), confirming the critical role for Vav in RP mobilization. Furthermore, the mutant NMJs showed significantly reduced PTP (Fig. 6 D), implying that Vav mediates PTP by driving RP mobilization.

**Figure 7.**
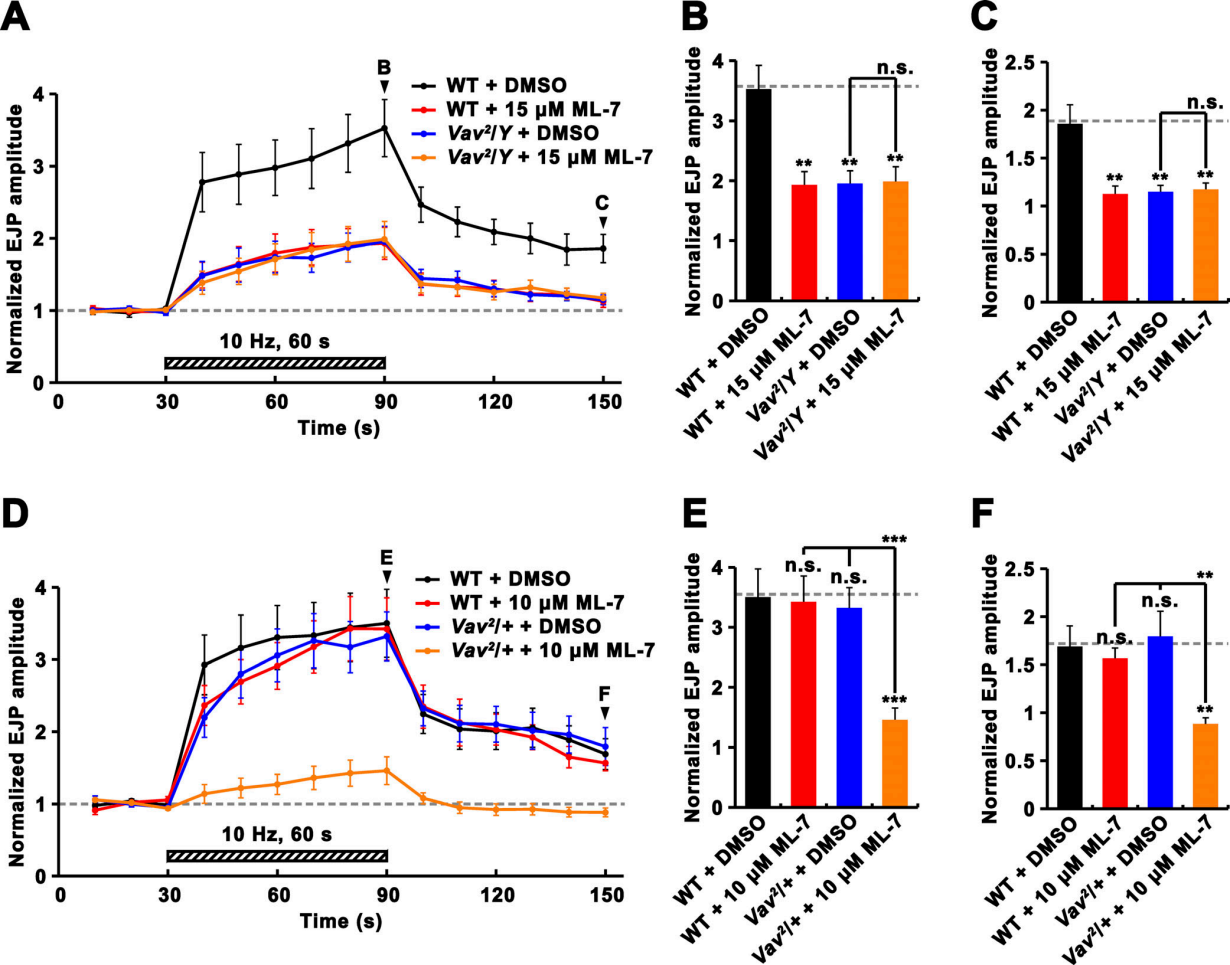
**Functional interaction between Vav and MLCK during PTP induction.** Larval preparations were preincubated in 0.3 mM Ca^2+^ saline with 0.1% DMSO or the MLCK inhibitor ML-7 for 30 min, and PTP was then induced using the 10 Hz, 1 min PTP induction protocol described in Fig. 4 A. **(A–C)** Pretreatment with 15 μM ML-7 decreases PTP in WT larvae but does not further decrease PTP in *Vav*^*2*^/*Y* mutant larvae. **(A)** Plot of mean EJP amplitudes normalized to mean initial amplitude before, during, and after PTP induction protocol. Each point in the ordinate represents the mean normalized amplitude for every 10 s. **(B and C)** Bar graphs of mean normalized EJP amplitudes for the 10-s period at the end of (B) and at 60 s after (C) tetanic stimulation. **(D–F)** Pretreatment with 10 μM ML-7, which has no effect on synaptic potentials in WT larvae, significantly impairs PTP in *Vav*^*2*^/+ heterozygotes. **(D)** Plot of mean normalized EJP amplitudes before, during, and after the PTP induction protocol. **(E and F)** Bar graphs of mean normalized EJP amplitudes at the end of (E) and at 60 s after (F) tetanic stimulation. Data represent mean ± SEM, *n* = 12 larvae. Comparisons are with WT (**, P < 0.01; ***, P < 0.001; n.s., not significant). Dashed lines represent mean pre- or post-tetanus WT + DMSO values.

To strengthen the above conclusion, we examined Vav’s functional link to MLCK during PTP induction. Pretreatment of WT larvae with the MLCK inhibitor ML-7 (15 μM) significantly inhibited synaptic augmentation and PTP (Fig. 7, A–C). However, ML-7 pretreatment had no effect on levels of synaptic augmentation and PTP in *Vav*^*2*^ mutants (Fig. 7, A–C), indicating that *Vav* and the MLCK inhibitor affect the same mechanism driving PTP. Furthermore, ML-7 at a lower concentration (10 μM) did not affect synaptic augmentation and PTP in WT larvae but decreased their levels in *Vav*^*2*^/+ heterozygotes (Fig. 7, D–F), confirming a functional link between Vav and MLCK during PTP induction. These findings are consistent with the model that Vav mediates PTP through RP mobilization.

### Vav acts in the Rac1-SCAR pathway to regulate synaptic plasticity

**Figure 8.**
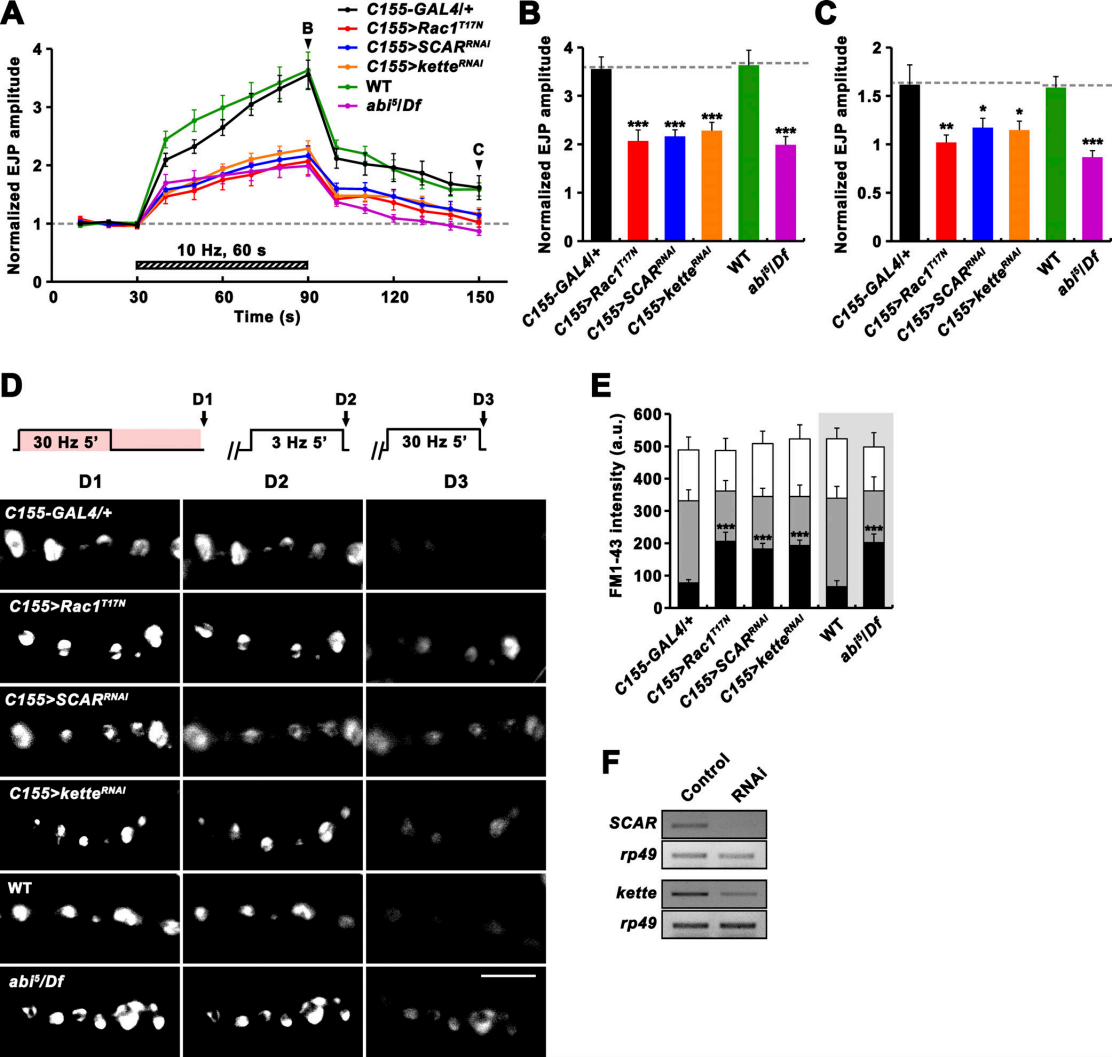
**Rac1-SCAR pathway components are required for PTP and RP mobilization. (A–C)** EJPs were recorded before, during, and after a 10 Hz, 1 min PTP induction protocol (0.3 mM Ca^2+^; Fig. 4 A) from *C155-GAL4*/+, *C155-GAL4*/+; *UAS-Rac1*^*T17N*^/+ (*C155>Rac1*^*T17N*^), *C155-GAL4*/+; *UAS-Dicer-2*/+; *UAS-SCAR*^*RNAi*^/+ (*C155>SCAR*^*RNAi*^), *C155-GAL4*/+; *UAS-Dicer-2*/+; *UAS-kette*^*RNAi*^/+ (*C155>kette*^*RNAi*^), WT, and *abi*^*5*^/*Df* larval preparations. **(A)** Plot of mean EJP amplitudes normalized to mean initial amplitude. Each point in the ordinate represents the mean normalized amplitude for every 10 s. **(B and C)** Bar graphs of mean normalized EJP amplitudes at the end of (B) and at 60 s after (C) tetanic stimulation. *n* = 15 larvae. Dashed lines represent mean *C155-GAL4*/+ or WT values. **(D)** After loading of ECP and RP with FM1-43 as described in Fig. 5 C, larval preparations of indicated genotypes were quickly washed with Ca^2+^-free saline and imaged (D1). Loaded preparations were subsequently stimulated at 3 Hz for 5 min in 2 mM Ca^2+^ saline without dye to unload ECP and imaged (D2). Preparations were further stimulated in 2 mM Ca^2+^ saline at 30 Hz for 5 min and reimaged (D3). **(E)** FM1-43 fluorescence intensity in boutons before (D1, height of whole columns) and after (D2, height of gray columns) 3 Hz stimulation, and after (D3, height of black columns) 30 Hz stimulation. Mean values were derived from 12 boutons from 6 different larvae. a.u., arbitrary units. **(F)** RT-PCR analysis of *SCAR* (two upper panels) and *kette* (two bottom panels) expression after ubiquitous expression of the *SCAR* or *kette* RNAi transgene (Control, *da-GAL4*/+; RNAi, *da-GAL4*>*SCAR*^*RNAi*^ or *kette*^*RNAi*^). *rp49* was used as a loading control. Data represent mean ± SEM. Statistically significant difference versus WT or *C155-GAL4*/+ is indicated (*, P < 0.05; **, P < 0.01; ***, P < 0.001). Scale bar: 5 μm. Source data are available for this figure: SourceData F8.

**Figure 9.**
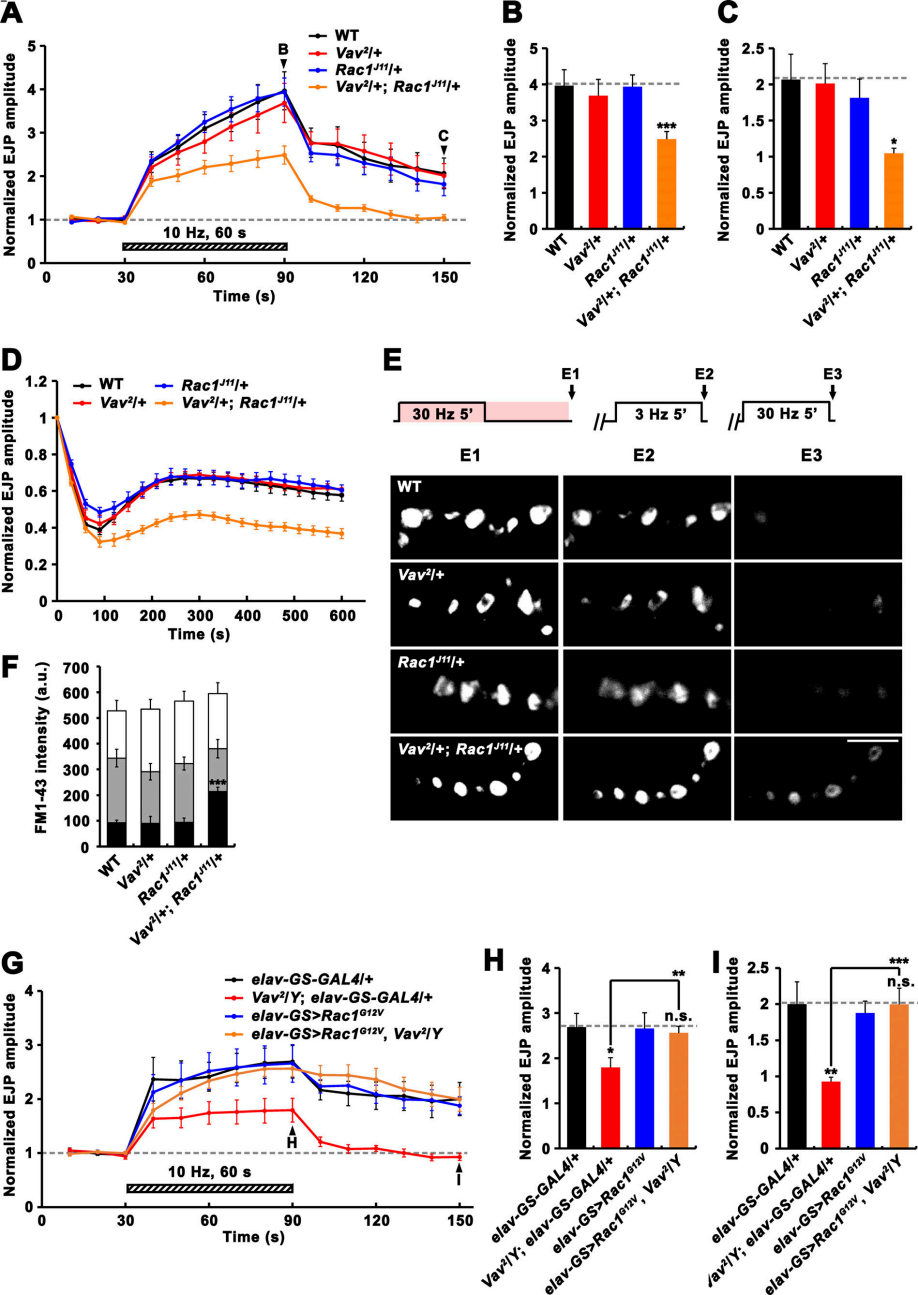
**Trans-heterozygous genetic interaction between *Vav* and *Rac1* during PTP induction, synaptic depression, and RP mobilization. (A–C)** Trans-heterozygous interaction between *Vav* and *Rac1* during PTP induction under low Ca^2+^. EJPs were recorded before, during, and after a 10 Hz, 1 min PTP induction protocol (0.3 mM Ca^2+^; Fig. 4 A) from WT, *Vav*^*2*^/+, *Rac1*^*J11*^/+, and *Vav*^*2*^/+; *Rac1*^*J11*^/+ larvae. **(A)** Plot of mean EJP amplitudes normalized to mean initial amplitude are shown over time for indicated genotypes. Each point in the ordinate represents the mean normalized amplitude for every 10 s. **(B and C)** Bar graphs of mean normalized EJP amplitudes at the end of (B) and at 60 s after (C) tetanic stimulation. *n* = 12 larvae. **(D)** Trans-heterozygous interaction between *Vav* and *Rac1* during synaptic depression under high Ca^2+^. Synaptic depression was induced using a 10 Hz, 10 min train in 10 mM Ca^2+^. Each point in the ordinate represents the mean normalized amplitude for every 30 s. *n* = 12 larvae. **(E and F)** Trans-heterozygous interaction between *Vav* and *Rac1* during tetanus-induced RP mobilization. **(E)** After loading of ECP and RP with FM1-43 as described in Fig. 5 C, larval preparations of indicated genotypes were quickly washed with Ca^2+^-free saline and imaged (E1). Loaded preparations were subsequently stimulated at 3 Hz for 5 min in 2 mM Ca^2+^ saline without dye to unload ECP and imaged (E2). Preparations were further stimulated in 2 mM Ca^2+^ saline at 30 Hz for 5 min and reimaged (E3). **(F)** FM1-43 fluorescence intensity in boutons before (E1, height of whole columns) and after (E2, height of gray columns) 3 Hz stimulation, and after (E3, height of black columns) 30 Hz stimulation. Mean values were derived from 12 boutons from six different larvae. a.u., arbitrary units. **(G–I)** The reduced PTP phenotype of *Vav* mutants is suppressed by neuronal overexpression of constitutively active Rac1 (Rac1^G12V^). EJPs were recorded before, during, and after a 10 Hz, 1 min PTP induction protocol (0.3 mM Ca^2+^; Fig. 4 A) from larvae of indicated genotypes. Animals were fed with RU486 during larval development. **(G)** Plot of mean EJP amplitudes normalized to mean initial amplitude. **(H and I)** Bar graphs of mean normalized EJP amplitudes at the end of (H) and at 60 s after (I) tetanic stimulation. *n* = 12 larvae. Data represent mean ± SEM. Dashed lines represent mean WT or *elav-GS-GAL4*/+ values. Statistically significant difference versus WT is indicated (*, P < 0.05; **, P < 0.01; ***, P < 0.001; n.s., not significant). Scale bar: 5 μm.

Having found that Vav plays important roles in RP mobilization and PTP, we next examined whether these tetanus-induced processes also require the Rac1-SCAR cascade. Neuronal expression of *Rac1*^*T17N*^, *SCAR*^*RNAi*^, or *kette*^*RNAi*^ impaired PTP and RP mobilization (Fig. 8). Comparable phenotypes were also observed in *abi* mutants (Fig. 8, A–E). In addition, trans-heterozygous genetic interaction between *Vav*^*2*^ and *Rac1*^*J11*^ was observed during PTP induction, synaptic depression, and RP mobilization (Fig. 9, A–F). Furthermore, neuronal overexpression of constitutively active Rac1 (Rac1^G12V^) throughout larval development, which had no effect in a WT background, completely suppressed the PTP defect observed in *Vav*^*2*^/*Y* mutants (Fig. 9, G–I). These results imply that Vav acts upstream of the actin-regulatory Rac1-SCAR pathway to regulate activity-dependent synaptic plasticity.

### Vav/Rac1-mediated regulation of actin dynamics plays a direct role in synaptic plasticity mechanism

**Figure S5.**
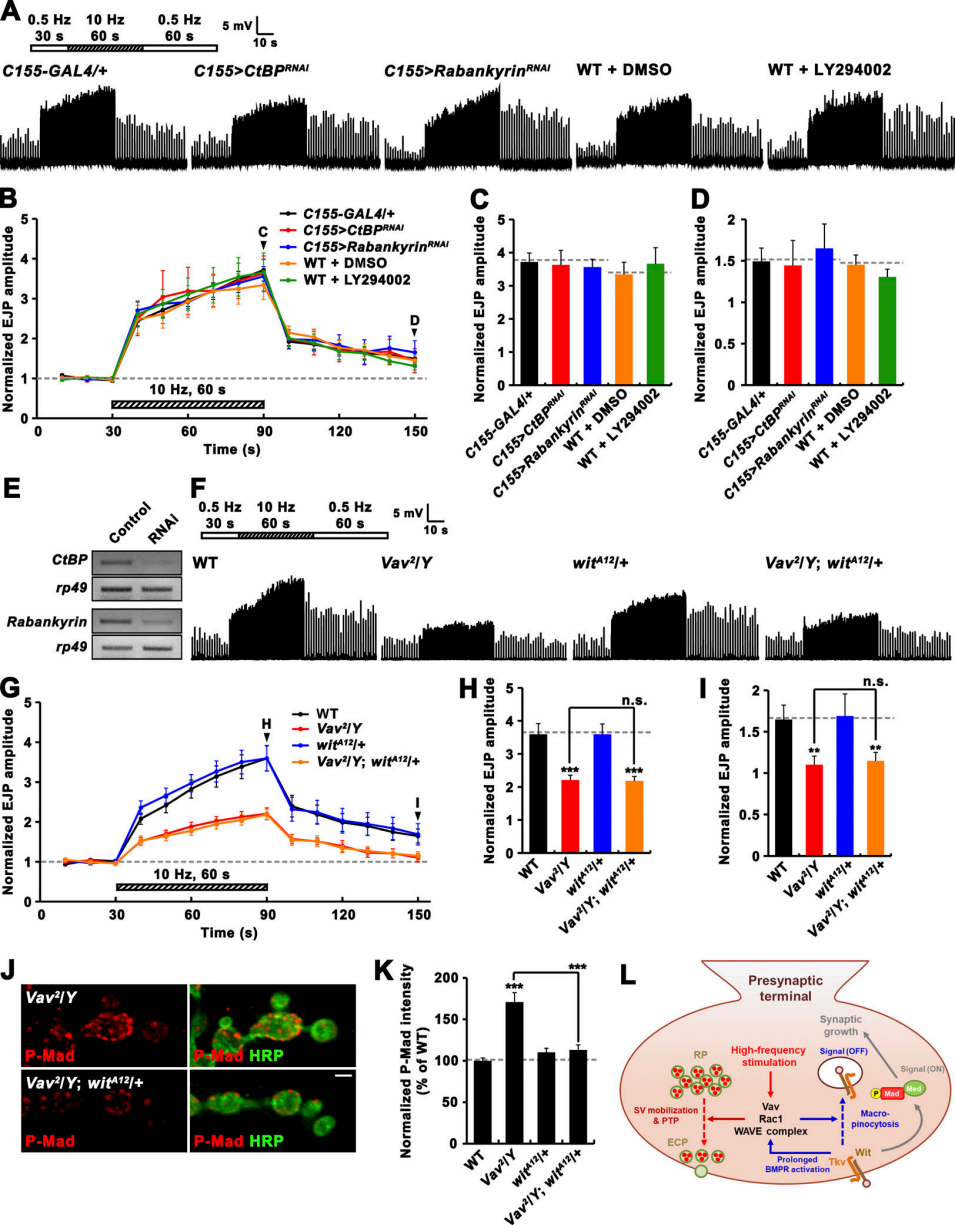
**Reduced PTP in *Vav* mutants is not secondary consequences of impaired macropinocytosis or excessive BMP signaling. (A–D)** PTP is not impaired by genetic or pharmacological perturbation of macropinocytosis. **(A)** Representative recordings from WT, *C155-GAL4*/+, *C155-GAL4*/+; *UAS-CtBP*^*RNAi*^/+ (*C155>CtBP*^*RNAi*^), *C155-GAL4*/+; *UAS-Rabankyrin*^*RNAi*^/+ (*C155>Rabankyrin*^*RNAi*^) larvae in 0.3 mM Ca^2+^ saline. The stimulation paradigm was 0.5 Hz for 30 s (white bar), 10 Hz for 60 s (hatched bar), and 0.5 Hz for the remainder of experiment (white bar). For WT larvae, filleted preparations were preincubated in 0.3 mM Ca^2+^ saline with vehicle alone (0.1% DMSO, WT + DMSO) or the macropinocytosis inhibitor (25 μM LY294002, WT + LY294002) for 30 min, prior to PTP experiments. **(B)** Plot of mean EJP amplitudes normalized to mean initial amplitude. Each point in the ordinate represents the mean normalized amplitude for every 10 s. **(C and D)** Bar graphs of mean normalized EJP amplitudes for the 10-s period right before (C) and after (D) cessation of tetanic stimulation. *n* = 12 larvae. **(E)** RT-PCR analysis of *CtBP* (two upper panels) and *Rabankyrin* (two bottom panels) expression after ubiquitous expression of the *CtBP* or *Rabankyrin* RNAi transgene (Control, *da-GAL4*/+; RNAi, *da-GAL4*>*CtBP*^*RNAi*^ or *Rabankyrin*^*RNAi*^). *rp49* was used as a loading control. **(F–K)** Removing one copy of *wit* in *Vav*^*2*^/*Y* mutants restores synaptic P-Mad, but not PTP, to WT levels. **(F)** Representative recordings from WT, *Vav*^*2*^/*Y*, *wit*^*A12*^/+, and *Vav*^*2*^/*Y*; *wit*^*A12*^/+ larvae in 0.3 mM Ca^2+^ saline. The stimulation paradigm was the same as in A. **(G)** Plot of mean EJP amplitudes normalized to mean initial amplitude. **(H and I)** Bar graphs of mean normalized EJP amplitudes for the 10-s period right before (H) and after (I) cessation of tetanic stimulation. *n* = 15 larvae. **(J)** Single confocal sections of NMJ 6/7 doubly labeled with anti–P-Mad (red) and anti-HRP (green) in WT, *Vav*^*2*^/*Y*, *wit*^*A12*^/+, and *Vav*^*2*^/*Y*; *wit*^*A12*^/+ larvae. **(K)** Quantification of the ratio of P-Mad to HRP intensities. Values are percentages of WT. *n* = 24 NMJs. (**L)** Model for Vav-Rac1 signaling-dependent regulation of synaptic growth and PTP. Data represent mean ± SEM. Comparisons are with WT (**, P < 0.01; ***, P < 0.001; n.s., not significant). Dashed lines represent mean *C155-GAL4*/+ or WT values. Scale bar: 2 μm. Source data are available for this figure: SourceData FS5.

Since the Vav-Rac1 pathway is also involved in synaptic macropinocytosis-dependent downregulation of BMP signaling implicated in activity-dependent synaptic plasticity (Berke et al., 2013), the PTP defect of *Vav* mutants may arise from impaired synaptic macropinocytosis or elevated BMP signaling. To test this, we first examined if interfering with macropinocytosis inhibits PTP induction (Fig. S5, A–E). We found that synaptic augmentation and PTP were not altered by neuronal depletion of CtBP or Rabankyrin, two well-established regulators of synaptic macropinocytosis. Furthermore, there were no abnormalities in synaptic augmentation and PTP when WT preparations were pretreated with the macropinocytosis inhibitor LY294002. Next, we tested if reduction of *wit* function suppresses the PTP phenotype of *Vav* mutants with elevated BMP signaling activity. When one copy of *wit* was removed in the *Vav*^*2*^ mutant background, there was a PTP defect comparable to that in *Vav*^*2*^ mutants (Fig. S5, F–I). In contrast, heterozygosity for *wit* in *Vav*^*2*^ mutants completely rescued elevated P-Mad levels and synaptic overgrowth at the NMJ (Fig. 2 B; and Fig. S5, J and K). These results support that PTP and macropinocytosis/BMP signaling are independently regulated by the Vav-Rac1 pathway.

**Figure 10.**
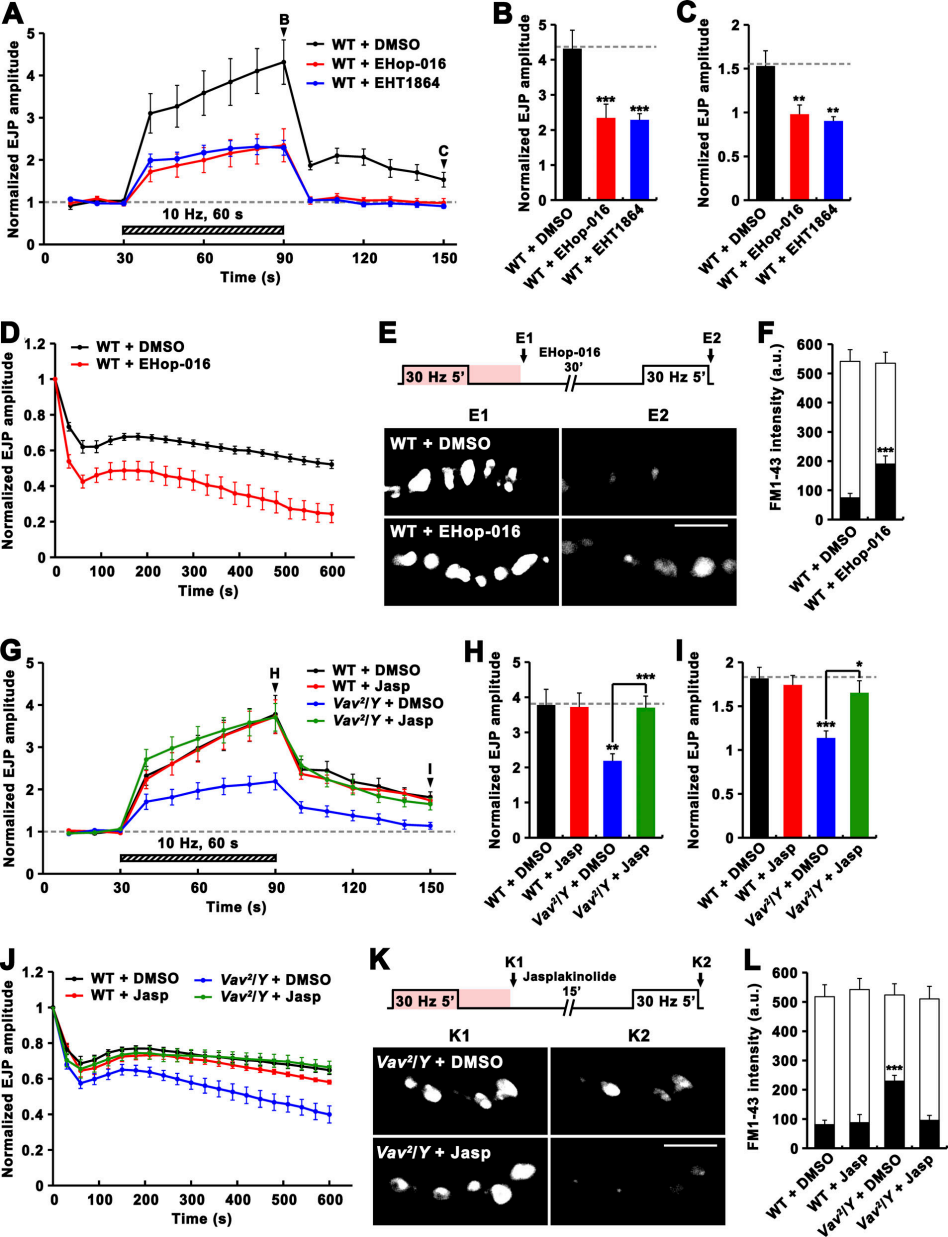
**Regulation of F-actin dynamics by Vav-Rac1 signaling is required for activity-dependent synaptic plasticity and RP mobilization. (A–F)** Acute blockade of Vav-Rac1 signaling phenocopies the defects of *Vav* or *Rac1* loss-of-function mutants in tetanus-induced synaptic plasticity and RP mobilization. **(A–C)** Effects of acute Vav-Rac1 blockade on synaptic augmentation and PTP. WT larval preparations were preincubated for 30 min in 0.3 mM Ca^2+^ saline containing an inhibitor of Vav-Rac1 signaling (20 μM EHop-016 or 50 μM EHT 1864 in 0.1% DMSO), and PTP was assayed using the 10 Hz, 1 min PTP induction protocol. **(A)** Plot of mean EJP amplitudes normalized to mean initial amplitude. Each point in the ordinate represents the mean normalized amplitude for every 10 s. **(B and C)** Bar graphs of mean normalized EJP amplitudes at the end of (B) and at 60 s after (C) tetanic stimulation. *n* = 12 larvae. **(D)** Effect of EHop-016 on synaptic depression. Plot of mean EJP amplitudes normalized to mean initial amplitude is shown for 0.1% DMSO- or EHop-016–treated WT larvae. Synaptic depression was induced using a 10 Hz, 10 min train in 10 mM Ca^2+^. Each point in the ordinate represents the mean normalized amplitude for every 30 s. *n* = 12 larvae. **(E and F)** Effect of EHop-016 on RP mobilization. **(E)** WT boutons loaded with FM1-43 using the ECP-RP loading protocol as described in Fig. 5 C were quickly washed with Ca^2+^-free saline and imaged (E1). RP-loaded boutons were subsequently stimulated at 30 Hz for 5 min and reimaged (E2). **(F)** FM1-43 fluorescence intensity in boutons before (height of whole columns) and after (height of black columns) 30 Hz stimulation. *n* = 12 boutons from six different larvae. **(G–L)** Application of jasplakinolide rescues the defects of *Vav* mutants in tetanus-induced synaptic plasticity and RP mobilization. Experimental paradigms and data presentation are the same as in A–F except that WT and *Vav*^*2*^*/Y* larval preparations were preincubated with 10 μM jasplakinolide in 0.1% DMSO. **(G–I)** Rescue of reduced PTP in *Vav*^*2*^*/Y* mutants by jasplakinolide. *n* = 15 larvae. **(J)** Rescue of enhanced synaptic depression in *Vav*^*2*^*/Y* mutants by jasplakinolide. *n* = 12 larvae. **(K and L)** Rescue of reduced RP mobilization in *Vav*^*2*^*/Y* mutants by jasplakinolide. *n* = 12 boutons from six different larvae. Data represent mean ± SEM. Comparisons are with WT (*, P < 0.05; **, P < 0.01, ***, P < 0.001). Dashed lines represent mean pre- or post-tetanic WT + DMSO values. Scale bars: 5 μm.

Given this conclusion and the previously demonstrated role of F-actin in RP mobilization (Delgado et al., 2000), another possibility is that the Vav-Rac1 pathway plays a direct role in synaptic plasticity mechanisms through acute regulation of actin cytoskeletal dynamics. To test this, we first investigated the effects of acutely blocking Vav-mediated Rac1 activation on tetanus-induced synaptic plasticity and RP mobilization. We pretreated WT larval preparations for 30 min with EHop-016 (20 μM), a drug that specifically prevents Vav-mediated Rac1 activation (Montalvo-Ortiz et al., 2012), and repeated PTP, depression, and RP mobilization assays in the continued presence of the drug. Treatment with EHop-016 abolished PTP under low Ca^2+^ and enhanced synaptic depression under high Ca^2+^ (Fig. 10, A–D). Moreover, tetanus-induced RP mobilization was inhibited by EHop-016 (Fig. 10, E and F). Comparable phenotypes were also observed in preparations treated with 50 μM EHT 1864 (Fig. 10, A–C), a drug placing Rac1 in an inert and inactive state (Onesto et al., 2008). Thus, acute application of Vav/Rac1 inhibitors phenocopies *Vav* or *Rac1* loss-of-function mutants, supporting a direct role of Vav-Rac1 signaling in activity-dependent synaptic plasticity and RP mobilization. Next, we tested whether the functional synaptic defects of *Vav* mutants can be alleviated by jasplakinolide. Pretreatment with jasplakinolide (10 μM) completely rescued reduced PTP, enhanced synaptic depression, and reduced RP mobilization in *Vav*^*2*^ mutants, and had no effect on WT (Fig. 10, G–L). These data demonstrate an important role of Vav-mediated actin polymerization in RP mobilization and highlight a cellular mechanism underlying tetanus-induced synaptic plasticity.

## Discussion

Modulation of presynaptic actin dynamics is thought to underlie synapse morphogenesis, endocytosis, SV organization and mobilization, and other cellular processes relevant to synaptic growth and function (Cingolani and Goda, 2008; Papandréou and Leterrier, 2018; Rust and Maritzen, 2015). However, little is known about the molecular mechanisms that regulate presynaptic actin dynamics in such processes. In addition, it remains unclear to what extent actin-based cellular processes affecting synaptic structure and function are independently regulated. Here, we present data indicating that the *Drosophila* Vav GEF acts upstream of the actin-regulatory Rac1-SCAR pathway to regulate BMP-dependent synaptic growth and activity-dependent plasticity via distinct cellular mechanisms, both of which require modulation of presynaptic actin dynamics.

*Drosophila* and mammalian Vav GEFs have been implicated in multiple aspects of nervous system development, including neuronal migration as well as axon growth and guidance (Aoki et al., 2005; Cowan et al., 2005; Malartre et al., 2010; Sauzeau et al., 2010; Schmid et al., 2004). Furthermore, mammalian Vav proteins have been shown to mediate brain-derived neurotrophic factor-induced dendritic spine head growth and theta burst-stimulated long-term potentiation in the hippocampus (Hale et al., 2011), suggesting the role of Vav GEFs in regulating postsynaptic structure and function. In this study, we provide the first evidence for Vav’s presynaptic role at the *Drosophila* NMJ, where the retrograde BMP signaling cascade constitutes a key signal promoting synaptic growth (Aberle et al., 2002; Marques et al., 2002; McCabe et al., 2003; Rawson et al., 2003; Sweeney and Davis, 2002). Our genetic data imply that Vav acts as a presynaptic negative regulator of retrograde BMP signaling. First, loss-of-function mutations in *Vav* cause synaptic overgrowth with excessive satellite bouton formation, as do mutations causing abnormal elevation of BMP signaling; our rescue experiments show that Vav acts pre-synaptically to regulate synaptic growth. Second, elevated levels of the BMP receptor Tkv and P-Mad are observed at *Vav* mutant NMJs. Third, genetic interaction experiments indicate that synaptic overgrowth in *Vav* depends on the activity of BMP signaling. Our data suggest that Vav restrains synaptic growth by antagonizing presynaptic BMP signaling.

Our previous report provided insights into the important contribution of Rac1/SCAR-mediated macropinocytosis to ligand-induced endocytic downregulation of presynaptic BMPRs (Kim et al., 2019). Our current findings imply a functional link between Vav and Rac1/SCAR in this process. First, GEF-defective Vav-L443A fails to rescue synaptic overgrowth in *Vav* mutants, suggesting that Vav protein exerts its effect on synaptic growth by triggering activation of Rho GTPases through GEF activity. Second, Vav is necessary for BMP-induced macropinocytosis. Third, trans-heterozygous interaction between *Vav* and a key component of macropinocytosis (*CtBP*) suggests that they function in the same cellular process to regulate synaptic growth. Finally, further genetic-interaction experiments reveal that Vav acts as an upstream regulator of the Rac1-SCAR pathway during synaptic growth.

*Vav* mutant NMJs display normal synaptic transmission under low-frequency (0.5 Hz) stimulation. However, they exhibit abnormalities in two forms of short-term plasticity induced by prolonged high-frequency (10 Hz) stimulation: reduced PTP under low Ca^2+^ and enhanced depression under high Ca^2+^. Neuronal expression of Vav completely rescues these mutant phenotypes, revealing its presynaptic role in functional synaptic plasticity. However, the GEF-defective Vav-L443A mutant fails to rescue both reduced PTP and enhanced depression phenotypes, suggesting the involvement of the Rac1-SCAR pathway. Consistently, loss of Rac1 or a SCAR complex component (Kette or SCAR) also impairs PTP, and *Vav* and *Rac1* display trans-heterozygous interaction to produce the *Vav*-like phenotypes of reduced PTP and enhanced depression. The loss of Cyfip, another component of the SCAR complex, enhances synaptic depression during tetanic stimulation under high Ca^2+^ (Zhao et al., 2013), further supporting a functional link between Vav and the Rac1-SCAR pathway during activity-dependent synaptic plasticity.

How might *Vav* mutations selectively impair tetanus-induced plasticity but not basal transmission? During low-frequency stimulation, the readily releasable pool (RRP) is maintained by endocytosis of recently released vesicles. However, at high stimulation frequencies, the RRP should be additionally replenished by recruitment of vesicles from the RP to sustain synaptic transmission and to induce PTP (Kim et al., 2009). Therefore, it is highly tempting to speculate that alterations in tetanus-induced plasticity in *Vav* mutants may be caused by defects in SV dynamics. Consistent with this hypothesis, our electrophysiological and FM1-43 labeling experiments reveal severely impaired RP mobilization in *Vav* mutants. However, loss of Vav does not change the size and endocytosis-mediated replenishment of the RP and ECP, which is closely correlated with the RRP at the *Drosophila* NMJ (Delgado et al., 2000), supporting the specific role of Vav in RP mobilization.

RP mobilization is disrupted by the F-actin-destabilizing drug cytochalasin D (Delgado et al., 2000; Kuromi and Kidokoro, 1998) or inhibitors of MLCK (Ryan, 1999; Verstreken et al., 2005), which is a major activator of the actin-based motor protein myosin. These studies suggest a central role of actin polymerization in RP recruitment. Our data indicate that Vav is involved in this regulatory process. Our approach using the MLCK inhibitor ML-7 reveals a functional interaction between *Vav* and MLCK. We also find that treatment of *Vav* mutant NMJs with the F-actin stabilizer jasplakinolide completely rescues defects in RP mobilization and restores alterations in PTP and tetanus-induced depression. These findings support the model that Vav drives activity-dependent plasticity by promoting RP mobilization through actin polymerization.

An important caveat to this conclusion, however, is that *Vav* mutations also elevate levels of BMP signaling, leading to chronic changes in target gene expression during development. In addition, the effects of macropinocytosis itself on synaptic plasticity have not been investigated. Therefore, the effect of Vav loss on synaptic plasticity may arise as a secondary consequence of elevated BMP signaling and defective macropinocytosis. Several experiments argue against these alternative possibilities. First, we demonstrate that genetic or pharmacological disruption of macropinocytosis does not impair PTP. Next, the severity of the PTP phenotype is not significantly different between *Vav* mutants and *Vav* mutants carrying a heterozygous *wit* mutation, which restores the level of BMP signaling to WT. Finally, acute inhibition of the Vav-Rac1 pathway efficiently reduces RP mobilization, causing alterations in tetanus-induced plasticity: reduced PTP under low Ca^2+^ and enhanced depression under high Ca^2+^. Based on these observations, we propose that the actin-regulatory Vav-Rac1 pathway plays a direct role in mechanisms of synaptic plasticity.

A final point of interest is related to the spatial control of Vav signaling activity at the presynaptic terminal. Although actin filament networks are generally known to play multiple roles in SV exocytosis and endocytosis (Wu and Chan, 2022), loss of Vav activity specifically impairs BMP-induced synaptic macropinocytosis but not SV cycling associated with basal neurotransmission. Furthermore, Vav-mediated actin polymerization is required for tetanus-induced mobilization of RP vesicles located away from the presynaptic membrane. Since the roles of Vav in synaptic macropinocytosis and RP mobilization are genetically separable, we propose a model in which the activation or subcellular localization of Vav is differentially regulated in different subcellular compartments to fine-tune the dynamics of distinct actin pools with highly specialized tasks during synaptic plasticity (Fig. S5 L). A future challenge will be to dissect the mechanisms by which synaptic growth signals and repetitive neuronal firing achieve distinct local activation of Vav within the presynaptic compartment. This study will advance our understanding of the regulation of presynaptic structural and functional plasticity by local actin dynamics.

## Materials and methods

### *Drosophila* stocks

The *w*^*1118*^ strain was used as the WT control. The following fly lines were generously provided by M. Martin-Bermudo (Malartre et al., 2010; University Pablo de Olavide, Sevila, Spain): *Vav*^*2*^, *Vav*^*3*^, and *UAS-Vav*^*CA*^*-HA*. Transgenic flies carrying *Vav-HA*, *Vav-L443A-HA*, and *UAS-Myc-GluRIIC-Flag* were generated in the *w*^*1118*^ background by standard injection methods (BestGene). The *abi*^*5*^ and *UAS-Myc-tkv* lines have been previously described (Kim et al., 2019). The following lines were obtained from the Bloomington *Drosophila* Stock Center: *wit*^*A12*^ (Marques et al., 2002), *wit*^*B11*^ (Marques et al., 2002), *dad*^*j1E4*^, *Rac1*^*J11*^ (Ng and Luo, 2004), *SCAR*^*Δ37*^ (Zallen et al., 2002), *kette*^*J4-48*^ (Hummel et al., 2000), *CtBP*^*03463*^, *UAS-Rac1*^*G12V*^ (Luo et al., 1994), *UAS-Rac1*^*T17N*^ (Luo et al., 1994), *Df(3R)su(Hw)7*, *UAS-kette*^*RNAi*^, *UAS-SCAR*^*RNAi*^, *UAS-Rabankyrin*^*RNAi*^, and *UAS-CtBP*^*RNAi*^. The following GAL4 drivers were used to drive UAS transgenes: *C155-GAL4* (Lin and Goodman, 1994), *BG57-GAL4* (Budnik et al., 1996), *elav-GS-GAL4* (Osterwalder et al., 2001), *da-GAL4* (Wodarz et al., 1995), and *C155-GAL4*; *UAS-Dicer-2*.

Flies were maintained at 25°C on standard cornmeal medium. For Gene-Switch or jasplakinolide feeding experiments, embryos of various genotypes were collected on grape juice plates with yeast paste at the center. Collected embryos were placed on standard medium containing 10 μg/ml RU486 (Mifepristone; Sigma-Aldrich) or 10 μM jasplakinolide (Invitrogen) and developed until the third instar stage. Female animals were used for all experiments except those involving hemizygous *Vav* males in Figs. 1 A, and B, 2, 4, 5, 6, 7, A–C, 9, G–I, 10, G–L; and Figs. S1, S2, E and F, S3, S4, and S5, F–K.

### Molecular biology

A cDNA encoding the WT Vav with a C-terminal HA epitope (amino acid sequence YPYDVPDYA) was amplified as an EcoRI-XhoI fragment by PCR using the LD25754 cDNA template (Drosophila Genomics Resource Center) and then directly cloned into pTOP Blunt (Enzynomics) to generate *pTOP-Vav-HA*. The following primers were used to amplify the *Vav-HA* cDNA: 5′-GAA​TTC​GCC​ACC​ATG​GCC​AGC​AGC​AGT​AGC-3′ and 5′-CTC​GAG​TCA​AGC​GTA​ATC​TGG​AAC​ATC​GTA​TGG​GTA​AAG​CTC​TTC​GCT​GGC​C-3′. The L443A mutation was introduced into *pTOP-Vav-HA* via two-step PCR-based mutagenesis using the following primers: 5′-CTG​GAC​GTT​GCC​ACT​GCG​CTG​AAG​ACC-3′ and 5′-GGT​CTT​CAG​CGC​AGT​GGC​AAC​GTC​CAG-3′. For transgenic rescue experiments, *Vav-HA* and *Vav-L443A-HA* inserts were subcloned into the EcoRI/XhoI sites of pUAST (Brand and Perrimon, 1993) to produce *pUAS-Vav-HA* and *pUAS-Vav-L443A-HA*. For expression of Vav-GFP in BG2-c2 cells, *pAc-Vav-GFP* was generated by amplifying the *Vav* cDNA from the LD25754 clone and then direct cloning into the EcoRI/XhoI sites of *pAc5.1-GFP*, a derivative of *pAc5.1* (Invitrogen). The following primers were used to amplify the *Vav* cDNA: 5′-GAA​TTC​GCC​ACC​ATG​GCC​AGC​AGC​AGT​AGC-3′ and 5′-CTC​GAG​AAG​CTC​TTC​GCT​GGC​C-3′.

For Vav depletion in BG2-c2 cells, double-stranded RNA (dsRNA) for *Vav* was synthesized by in vitro transcription of a DNA template using the MEGAscript T7 Transcription kit (Invitrogen), as done previously (Kim et al., 2019). The DNA template was generated by PCR on the LD25754 cDNA template using primers containing T7 promoter sequence upstream of the following *Vav*-specific sequences: 5′-ACT​GAC​TGC​CAG​GTG​CTG​GTC​ATT​GGC-3′ and 5′-ACA​CTC​AGA​TTT​ATA​TAT​TTG​CAA​TAT-3′. The efficiency of *Vav* knockdown was assessed by RT-PCR analysis of total RNA extracted from BG2-c2 cells. The following primers were used for PCR reactions: *Vav*, 5′-GAG​TAT​GCT​CTT​CCT​CTT​CG-3′ and 5′-CAC​TGC​GAG​ATG​GCC​AGC​AG-3′; *rp49*, 5′-CAC​CAG​TCG​GAT​CGA​TAT​GC-3′ and 5′-CAC​GTT​GTG​CAC​CAG​GAA​CT-3′.

### Generation of endogenously HA-tagged Vav line

A C-terminal HA-tagged knock-in allele of *Vav* was generated by CRISPR/Cas9 system as described (Gratz et al., 2013). The gRNA target covering the *Vav* stop codon was generated by annealing the following complementary oligonucleotides: 5′-CTT​CGC​TTT​GAT​ATT​ACA​ACT​ACG-3′ and 5′-AAA​CCG​TAG​TTG​TAA​TAT​CAA​AGC-3′ and inserted into the BbsI site of the pU6-BbsI-chiRNA vector (Addgene). The following single-strand oligodeoxynucleotide (ssODN) was used: 5′-GGC​TAC​TTT​CCC​AAG​GAG​TAT​GTG​CAG​GAG​CAG​AAA​TTG​GCC​AGC​GAA​GAG​CT*TTA​CCC​TAC​GAT​GTT​CCA​GAT​TAC​GCT***TAA**T**TAA**T**TAA**CTA​CGA​GGT​TTA​CTT​TGC​ACC​CAA​GGC​CAT​TAC​GCC​CAC​AGC​GGC​AGC​CAT​TGC​TGA​ATT​GC-3′. The HA epitope tag and three consecutive stop codons are indicated in italics and bold, respectively. The gRNA plasmid and the ssODN were co-injected into *vas-Cas9*-expressing *Drosophila* embryos (#54591; Bloomington *Drosophila* Stock Center) by standard injection methods (BestGene).

### Cell line and transfection

*Drosophila* neuronal BG2-c2 (ML-DmBG2-c2) cells were obtained from the *Drosophila* Genomics Resource Center and maintained in Shields and Sang M3 insect medium (Sigma-Aldrich) supplemented with 10% heat-inactivated FBS, 0.5 mg/ml of KHCO_3_, 1 mg/ml yeast extract (BD Biosciences), 2.5 mg/ml bactopeptone (BD Biosciences), 10 μg/ml of insulin (Sigma-Aldrich), and an antibiotic mix of penicillin (60 μg/ml) and streptomycin (100 μg/ml; Welgene). Cells were transiently transfected with dsRNA or DNA constructs in serum-free M3 medium using Cellfectin II (Gibco) according to the manufacturer’s instructions.

### Live-cell imaging and macropinocytosis assay

To analyze association of Vav with macropinocytic structures, such as membrane ruffles and macropinocytic cups, BG2-c2 cells were transiently transfected with *pAc-Vav-GFP*, together with *pAc-PLC-PH-mCherry* (Kim et al., 2019), an initial macropinocytic marker. Transfected cells were transferred to a confocal culture dish (SPL Life Sciences) and serum-starved for 6 h. Culture medium was replaced with control or Gbb-conditioned medium just before initiating time-lapse imaging. Gbb-conditioned medium was prepared as previously described (Kim et al., 2019). Time-lapse images were acquired with a Zeiss LSM 800 confocal microscope using a Plan-Apo 100×/1.40 oil objective.

Macropinocytosis assays in BG2-c2 cells and larval NMJ preparations were performed as previously described (Kim et al., 2019). Briefly, mock- and *Vav* dsRNA-transfected cells were serum-starved for 6 h and then pulsed with 2 mg/ml of 70 kD TMR-Dextran (Invitrogen) in control-conditioned medium or Gbb-conditioned medium (50 ng/ml Gbb) for 5 min. Pulsed cells were fixed in 4% formaldehyde/PBS for 10 min and stained with PBS containing 1 μg/ml of DAPI (Invitrogen) for 10 min. Larval NMJ preparations were also treated with TMR-Dex in Gbb-conditioned medium as described above and fixed in 4% formaldehyde/PBS for 20 min. The fixed samples were further processed for FITC-HRP labeling to visualize NMJs as described below. A z-stack of optical sections was acquired with the Zeiss LSM 800 using a Plan-Apo 63×/1.25 oil objective, and maximum-intensity projection images were used to quantify the number of TMR-positive puncta (>0.2 μm in diameter) per cell or three terminal boutons at each NMJ branch.

### Immunohistochemistry and imaging of larval tissues

Wandering third instar larvae were dissected in ice-cold Ca^2+^-free HL3 saline (70 mM NaCl, 5 mM KCl, 20 mM MgCl_2_, 10 mM NaHCO_3_, 115 mM sucrose, 5 mM trehalose, 5 mM Hepes, pH 7.2). The larval fillets were fixed in either 4% formaldehyde/PBS for 30 min or Bouin’s solution (Sigma-Aldrich) for 10 min. Fixed larval fillets were washed three times with PBS containing 0.1% Triton X-100 (PBST-0.1) for 10 min and then incubated overnight at 4°C in 0.2% BSA/PBST-0.1 containing primary antibodies. Surface Myc-GluRIIC was stained in non-permeant conditions (Fig. S1). The following primary antibodies were used in this study: mouse anti-Brp (nc82; DSHB) at 1:10, mouse anti-Dlg (4F3; DSHB) at 1:200, mouse anti-CSP (1G12; DSHB) at 1:200, mouse anti-Syt (3H2 2D7; DSHB) at 1:5, rabbit anti-Myc (Cell Signaling Technology) at 1:200, rabbit anti-HA (Cell Signaling Technology) at 1:200, rabbit anti-P-Mad (PS1; (Persson et al., 1998) at 1:500, FITC-conjugated goat anti-HRP (Jackson ImmunoResearch Laboratories) at 1:200, and Alexa Fluor 647–conjugated goat anti-HRP (Jackson ImmunoResearch Laboratories) at 1:200. The samples were washed three times with PBST-0.1 and then incubated for 2 h at RT in 0.2% BSA/PBST-0.1 containing secondary antibodies. FITC-, Cy3-, and Alexa 647–conjugated secondary antibodies (Jackson ImmunoResearch Laboratories) were used at 1:200. Alexa 555–conjugated secondary antibodies (Invitrogen) were also used at 1:200. Images of NMJs were acquired with the Zeiss LSM 800 using a C-Apo 40×/1.2 W (for quantification of bouton number) or Plan-Apo 63 × 1.25 oil objectives. For quantification of bouton number, z-stack images for the entire NMJ were collected with 1 μm spacing, and maximum-intensity projection images were reconstructed using the Zen 3.4 software (Zeiss). Satellite boutons were determined as a single bouton that was not included in a chain of boutons. For quantification of Brp, P-Mad, and Myc-GluRIIC fluorescence intensities at the NMJ, optical sections through the middle of synaptic boutons were acquired.

Ventral nerve cords (VNCs) were dissected out of third instar larvae, fixed in 4% formaldehyde/PBS, and incubated with primary and secondary antibodies in 0.2% BSA/PBST-0.3 (PBS containing 0.3% Triton X-100). The following antibodies were used: anti-Elav (7E8A10; DSHB) at 1:10, anti–P-Mad at 1:500, and Cy3-/FITC-conjugated secondary antibodies (Jackson ImmunoResearch Laboratories) at 1:200.

### EM

EM analysis was performed essentially as described (Kim et al., 2021; Yao et al., 2017). Briefly, wandering third instar larvae dissected in Ca^2+^-free HL3 solution were fixed in 4% paraformaldehyde/1% glutaraldehyde/0.1 M cacodylic acid (pH 7.2) at 4°C for 12 h. The samples were then rinsed in 0.1 M cacodylic acid (pH 7.2), post-fixed in 1% OsO_4_/0.1 M cacodylic acid at RT for 3 h, and subjected to 30–100% ethanol dehydration steps. Subsequently, the samples were processed in propylene, a mixture of propylene and resin, and pure resin and embedded in 100% resin. Imaging of NMJ boutons was performed using Tecnai G2 Spirit TWIN (FEI Company) and a Gatan CCD Camera (794.10.BP2 MultiScan) at ≥4,400× magnification. All data analyses were performed using ImageJ.

### Loading and unloading of FM1-43

Labeling of synaptic vesicles with FM1-43FX (Invitrogen) was achieved as previously described with slight modifications (Kuromi and Kidokoro, 2000). Briefly, wandering third-instar larvae were dissected in Ca^2+^-free HL3 saline. For loading synaptic vesicles with FM1-43FX, the nerve innervating muscle 6/7 in segment A3 was electrically stimulated in HL3 saline containing 2 mM Ca^2+^ and 4 μM dye as described in the figure legends. The preparations were washed three times with Ca^2+^-free HL3 saline and then imaged. For unloading FM1-43FX, nerves were restimulated in 2 mM Ca^2+^/HL3 at 3 or 30 Hz. Preparations were viewed and imaged on an upright fluorescence microscope (Axio Imager D1; Zeiss) equipped with Axiocam 506 monochrome camera and 63×/1.0 Plan-Apochromat water immersion objective. The average fluorescence intensities around individual boutons were calculated by subtracting the background fluorescence intensity using the Zen 3.4 software (Zeiss). Three type Ib boutons with an area >3 μm^2^ were selected for analysis in each preparation.

### Electrophysiology

For electrophysiological recordings, third instar larvae were dissected in Ca^2+^-free HL3 saline and briefly washed with modified HL3 saline (HL3 with MgCl_2_ reduced to 10 mM) with CaCl_2_ at concentrations specified in the figure legends. Intracellular recordings were taken from muscle 6 in segment A3 using microelectrodes (<25 MΩ) filled with 3 M KCl solution. Signals were amplified with Neuroprobe Amplifier (Model 1600; A-M Systems). EJP traces were analyzed using Clampfit software (version: 11.1; Molecular Devices). Analysis of more than 30 EJP events was performed using MATLAB R2020a (MathWorks). The amplitude and frequency of spontaneous mEJPs were analyzed using MiniAnalysis 6.0.7 (Synaptosoft). We only analyzed recordings from muscles with resting membrane potential below −65 mV and input resistance above 5 MΩ. Quantal content was calculated by dividing mean EJP by mean mEJP.

### Pharmacological reagents

NMJ preparations were pretreated with the following chemicals in some physiological recordings and FM1-43 labeling experiments: LY294002 (25 μM; Invitrogen), ML-7 (10 or 15 μM; Sigma-Aldrich), EHop-016 (20 μM; Sigma-Aldrich), EHT1864 (50 μM; Sigma-Aldrich), folimycin (1 μM; Sigma-Aldrich), and jasplakinolide (10 μM; Invitrogen). All chemicals were prepared as stocks in DMSO and diluted to desired concentrations in HL3 saline just before experiments. The final concentration of DMSO was kept below 0.1% (vol/vol) for all experiments.

### Estimation of vesicle pool size

The sizes of the cycling (or ECP) and total synaptic vesicle pools were determined as previously described with slight modifications (Kim et al., 2009). NMJ preparations were preincubated in HL3 saline with 1 μM folimycin and 2 mM Ca^2+^ for 5 min, and motor nerves were continuously stimulated at 3 Hz (for estimation of ECP size) or 10 Hz (for estimation of total vesicle pool size) for 15 min. Cumulative plots of quantal content versus time were created. Quantal content was corrected for non-linear summation of quanta at higher membrane potentials using a Martin correction factor. For estimation of ECP size, a line was fitted to points between 400 and 600 s and back-extrapolated to time 0. For estimation of total vesicle pool size, the cumulative quanta released during the 15-min 10 Hz train were determined.

### Statistical analysis

Data are presented as mean ± SEM. Unpaired Student’s *t*-test was used to compare two groups. For comparison of multiple groups, one-way ANOVA followed by Tukey’s post hoc test was conducted.

### Online supplemental material

Fig. S1 shows normal anatomical features of *Vav* mutant NMJs. Fig. S2 shows that Vav is required for Gbb-induced macropinocytosis. Fig. S3 shows that *Vav* mutants show normal evoked neurotransmission and presynaptic ultrastructure. Fig. S4 shows that the sizes of the ECP and the total vesicle pool in *Vav* mutants are normal. Fig. S5 shows that reduced PTP in *Vav* mutants is not a secondary consequence of impaired macropinocytosis or excessive BMP signaling.

## Supplementary Material

SourceData F8 contains original blots for Fig. 8.

SourceData FS2 contains original blots for Fig. S2.

SourceData FS5 contains original blots for Fig. S5.
