# Supplementary figures and images for: Vav independently regulates synaptic growth and plasticity through distinct actin-based processes
Source: J Cell Biol. 2022 Aug 17;221(10):e202203048. doi: 10.1083/jcb.202203048 (PMC9388202; doi:10.1083/jcb.202203048)

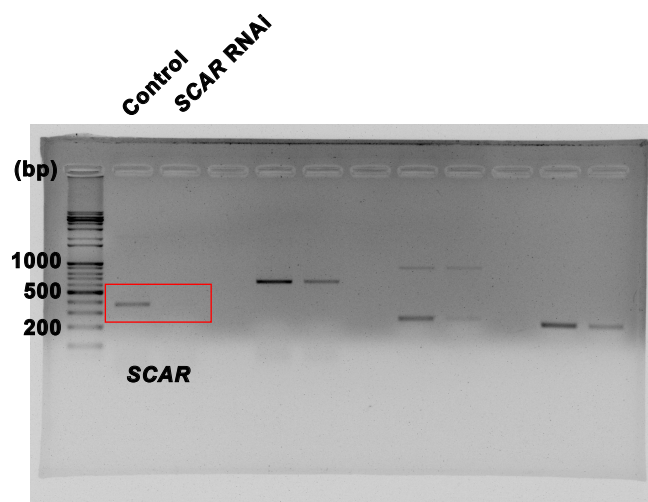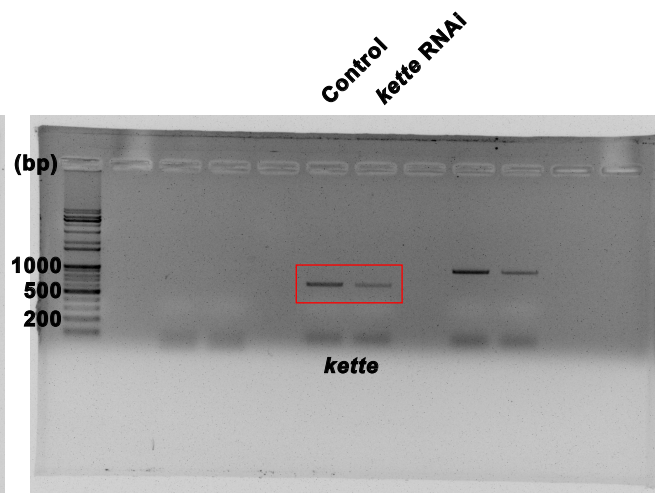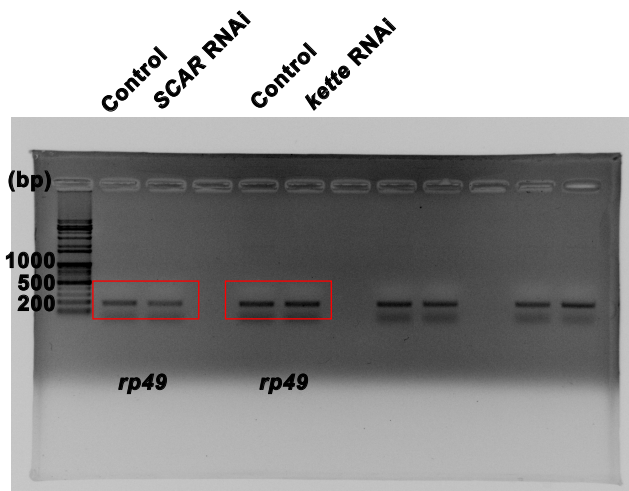

Supplement: SourceData F8 — contains original blots for Fig. 8. [file JCB_202203048_SourceDataF8.pdf]

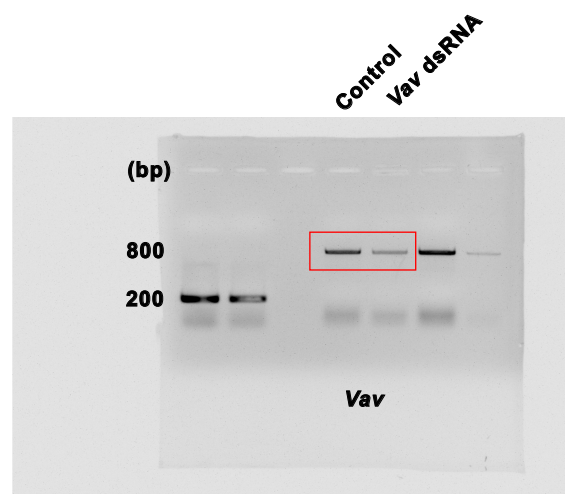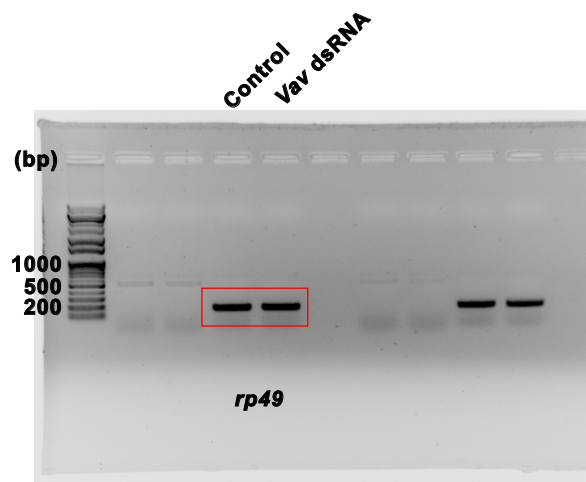

Supplement: SourceData FS2 — contains original blots for Fig. S2. [file JCB_202203048_SourceDataFS2.pdf]

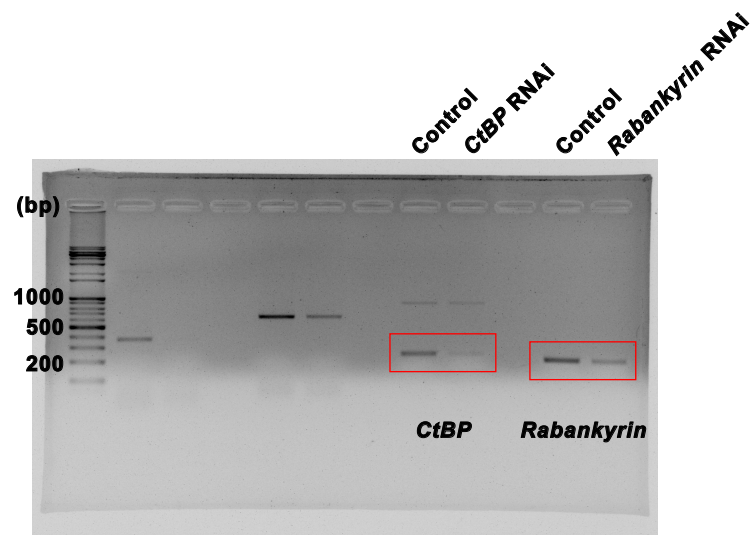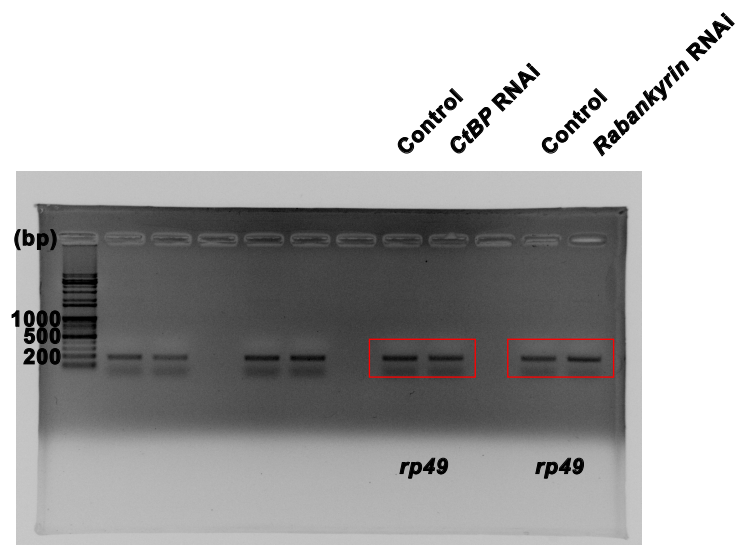

Supplement: SourceData FS5 — contains original blots for Fig. S5. [file JCB_202203048_SourceDataFS5.pdf]
